# Notch-Dependent Expression of the *Drosophila Hey* Gene Is Supported by a Pair of Enhancers with Overlapping Activities

**DOI:** 10.3390/genes15081071

**Published:** 2024-08-14

**Authors:** Maria Monastirioti, Ioanna Koltsaki, Ioanna Pitsidianaki, Emilia Skafida, Nikolaos Batsiotos, Christos Delidakis

**Affiliations:** 1 Institute of Molecular Biology and Biotechnology (IMBB), Foundation for Research and Technology-Hellas (FORTH), 70013 Heraklion, Greece; i.koltsaki@dkfz-heidelberg.de (I.K.); ioanna.pitsidianaki.20@ucl.ac.uk (I.P.); a.skafida@campus.unimib.it (E.S.); nickbatsi99@gmail.com (N.B.); 2 Department of Biology, University of Crete, 70013 Heraklion, Greece; 3 Division of Tumor Metabolism and Microenvironment, German Cancer Research Center (DKFZ), 69120 Heidelberg, Germany; 4 Department of Cell and Developmental Biology, University College London (UCL), London WC1E 6BT, UK; 5 Istituto di Ricovero e Cura a Carattere Scientifico (IRCCS), Foundation Saint Lucia, Rome and School of Medicine and Surgery, University of Milano-Bicocca (UniMiB), 20900 Monza, Italy; 6 Evotec SE, 22419 Hamburg, Germany

**Keywords:** Hey, basic helix–loop–helix–orange, Su(H) binding site, enhancer, *Drosophila*, Notch, central nervous system, midgut

## Abstract

*Drosophila* Hey is a basic helix–loop–helix–orange (bHLH-O) protein with an important role in the establishment of distinct identities of postmitotic cells. We have previously identified Hey as a transcriptional target and effector of Notch signalling during the asymmetric division of neuronal progenitors, generating neurons of two types, and we have shown that Notch-dependent expression of Hey also marks a subpopulation of the newborn enteroendocrine (EE) cells in the midgut primordium of the embryo. Here, we investigate the transcriptional regulation of *Hey* in neuronal and intestinal tissues. We isolated two genomic regions upstream of the promoter (HeyUP) and in the second intron (HeyIN2) of the *Hey* gene, based on the presence of binding motifs for Su(H), the transcription factor that mediates Notch activity. We found that both regions can direct the overlapping expression patterns of reporter transgenes recapitulating endogenous *Hey* expression. Moreover, we showed that while HeyIN2 represents a Notch-dependent enhancer, HeyUP confers both Notch-dependent and independent transcriptional regulation. We induced mutations that removed the Su(H) binding motifs in either region and then studied the enhancer functionality in the respective *Hey* mutant lines. Our results provide direct evidence that although both enhancers support Notch-dependent regulation of the *Hey* gene, their role is redundant, as a Hey loss-of-function lethal phenotype is observed only after deletion of all their Su(H) binding motifs by CRISPR/Cas9.

## 1. Introduction

Multiple signaling pathways as well as the transcriptional regulation of their effectors are important elements for the establishment of correct cell fates during embryonic development. The tissue and cell-specific as well as the temporal expression of genes involved in the above processes is controlled by multiple transcription factor binding sites found in their promoter regions and within *cis*-regulatory modules (CRMs), such as enhancers or silencers [1]. CRMs, which in several cases may exhibit overlapping spatiotemporal activity (e.g., shadow enhancers), are usually localized in the gene’s promoter upstream region, although they are often found in introns or in regions distal from the regulated gene and even within another gene locus [2].

The Notch signaling pathway has a central functional role in many of the early developmental processes and it can either promote or prevent cell differentiation [3,4,5]. 

Activation of the Notch pathway takes place upon interaction of the Notch receptor with its ligands, followed by the subsequent proteolytic cleavage and release of the Notch intracellular domain (N^icd^), which enters the nucleus and promotes a transcriptional activation response of the target genes. This is achieved by the interaction of N^icd^ with the DNA-binding proteins of the CSL family (for mammalian CBF1, *Drosophila* Suppressor of Hairless [Su(H)] and *Caenorhabditis elegans* Lag-1) that allows the recruitment of co-activators, such as Mastermind (Mam)/Mastermind-like (MAML) [6,7], reviewed in [8,9]. In the absence of Notch, CSL proteins can act as “default repressors”. *Drosophila* Su(H), bound to DNA, represses its targets by interacting with the adaptor protein Hairless (H) which recruits corepressors such as C-terminal-binding protein (CtBP), Groucho, Ski-interacting protein (Skip/Bx42) [10,11,12], and other protein complexes to establish a repressive chromatin state in the target promoters [13,14]. Upon Notch activation, Hairless and corepressors dissociate from Su(H) and are replaced by the N^icd^ domain which recruits the Mam coactivator and switches on the expression of target genes. 

The best-studied direct targets of Notch signaling are the genes of the Hairy/Enhancer of split (E(spl)) (Hes) family which encode bHLH-O transcriptional repressors, a class of bHLH transcription factors that are characterized by the “Orange” protein interaction domain and mediate much of the Notch pathway function (reviewed in [15]). *E(spl)* genes are strongly and quickly upregulated [16] in the majority of Notch-regulated processes, and their enhancers bear several Su(H) binding sites in single or paired (Su(H) paired site [SPS] configurations [17,18]. Several studies have reported that the disruption of such sites in the enhancer region of particular genes results in the abolishment of their expression, pointing to the importance of Su(H) binding for induction by Notch. At the same time, this disruption causes ectopic expression in cell types that do not receive Notch signaling, pointing to the importance of Su(H)-mediated default repression [17,18,19,20].

Another bHLH-O subfamily consists of the Hey proteins, which show high structural similarity with the Hes repressors. Hey proteins are named after Hes and a characteristic tyrosine residue in their C-terminus motif Tyr-Arg-Pro-Trp (YRPW) (Hes with a Y). Similarly to Hes genes, the three *Hey* paralogues (Hey1, Hey2 and HeyL) of the mammalian species and the single *Hey* gene found in *Drosophila* have been identified as direct targets of canonical Notch signaling which often transmit the Notch signal (reviewed in [21]). *Drosophila* Hey, in particular, has been detected in the young postmitotic neurons and few glia of the developing central nervous system (CNS) that receive a Notch signal at birth. The sibling cells arising from the division of the ganglion mother cell (GMC) precursor [22] adopt one of two alternative fates, “A” and “B”, which are regulated by different levels of Notch activity. Type “A” cells have high Notch activity, while in type “B” cells, there is no Notch activity [23,24]. Hey, which is transiently expressed in newly born type “A” neurons, is the first target of Notch signaling identified in this process and it is a sufficient effector of the pathway towards establishing the “A” fate [25,26]. Although in the majority of cases, Hey is a Notch target, its expression within the developing CNS displays Notch-independent domains as well, the most notable being the larva mushroom body (MB) lineages and cell groups along the lateral embryonic ventral nerve cord (VNC) [25]. In addition to the nervous system, *Hey* is also expressed in approximately half of the newly born enteroendocrine (EE) cells of the developing embryonic midgut primordium [27]. Its expression there is Notch-dependent and it presumably relates to the two different fates that are acquired by the EE cell population upon Notch signaling. On the other hand, in the adult midgut epithelium, Notch-independent low-level expression of Hey in the differentiated enterocytes (ECs) is critical for the maintenance of their cell identity [28]. 

Here we investigate the *Hey* genomic locus for regulatory enhancer regions that direct *Hey* gene expression in the developing neuronal and intestine tissues. The region upstream of the *Hey* transcription starting point and the second intron of the locus were identified as transcriptional CRMs driving reporter gene expression in spatiotemporally similar patterns which recapitulate Hey expression in both tissues studied. Both CRMs bear multiple Su(H) binding motifs for Notch-dependent activation, as validated by mutational or deletion analysis of the relevant sites with reporter transgenes and by monitoring the activity of each CRM in a Notch-deficient background. These experiments demonstrated the loss of the intronic enhancer activity, indicating that it is fully dependent on Notch and the residual activity of the upstream enhancer which, however, was detected mostly in Notch-independent sites of Hey expression. Finally, we generated three CRISPR (short for “clustered regularly interspaced short palindromic repeats”) mutants corresponding to deletions of the Su(H) sites in either enhancer or in both of them, as well as a deletion engineered by flippase/flippase recognition target (FLP/FRT) homologous recombination that removes the majority of the *Hey* locus and, therefore, represents a *Hey* null allele. By analyzing these mutants, we showed that there is full redundancy between the two CRMs with respect to their ability to activate normal Hey expression in response to Notch. Only the double-deletion CRISPR mutant exhibits a residual Hey presence, mostly at the sites of Notch-independent expression. Most importantly, this mutant is embryonic lethal, similarly to the *Hey* null allele, supporting the notion that the Notch-dependent component of Hey expression is required for embryo viability.

## 2. Materials and Methods

### 2.1. Fly Strains and Genetics

Flies were maintained under standard conditions at 25 °C, and the fly stocks used were generated during the study (Table 1) or were obtained from the Bloomington Drosophila Stock Center (https://bdsc.indiana.edu/ accessed on 2 July 2024).

For the integration of *attB* plasmids, stocks containing *attP40* (RRID: BDSC 25709) or *attP2* (RRID: BDSC 25710) docking sites were used, each carrying a chromosome expressing *ΦC31* integrase under the control of a *nanos* regulatory region [29]. For the generation of homozygous mutant *Dl^-^ Ser^-^* mosaic clones using the MARCM (short for ”Mosaic analysis with a repressible cell marker”) technique [30], the *FRT82B Dl^rev10^ e Ser^RX106^* chromosome (RRID: BDSC 6300 stock) was first combined with chromosomes bearing either of the HeyUP^FL^-bgal or HeyIN2-bgal reporter transgenes generated in this study. This was then crossed to the “MARCM 82B” strain, namely *hs-FLP atub-Gal4 UAS-GFP; FRT82B atub-Gal80.* For the FLP/FRT-based deletion [31] of the *Hey* locus, the Exelixis lines PBac{WH}f07552 (RRID:BDSC 85736) and PBac{WH}Hey[f06656] (RRID:BDSC 18997) [32] were combined via genetic crosses with an X chromosome bearing a heat-shock flippase transgene [P{hsFLP}]. The stock y^1^ M{nanos-Cas9.P}ZH-2A w* (RRID:BDSC 54591) [33], in which Cas9 is expressed in the germline cells under a nanos regulatory sequences, was injected with the gRNAs described in the study for the generation of CRISPR-induced *Hey* deletions. The enhancer trap line *AJ96-lacZ* was used to identify the dMP2 and vMP2 neurons of the median precursor 2 (MP2) lineage [34]. 

### 2.2. Immunohistochemistry

Fixation and subsequent immunohistochemistry of embryos and dissected larval tissues was performed according to standard protocols. The primary antibodies were rabbit anti-b-gal, 1:10,000 (Cappel Laboratories, Malvern, PA, USA); mouse anti-Eve (3C10), 1:30 (DSHB, Iowa City, IA, USA); mouse anti-Prospero (MR1A), 1:30 (DSHB); rabbit anti-GFP, 1:30,000 (Minotech Biotechnology, Heraklion, Greece); goat anti-GFP, 1:500 (Cat# AB0020-500, OriGeneTechnologies Inc., Rockville, MD, USA); mouse anti-Repo (8D14), 1:25 (DSHB); guinea pig anti-Hey 1:1000 [25]; Rabbit anti-Ase, 1:1000 (a gift from Claude Desplan, NYU Department of Biology, New York, NY, USA). Detection was performed using secondary antibodies conjugated to Alexa 488, 555, 568, 633, or 647 (Thermo Fisher Scientific Inc., Waltham, MA, USA), or Cy3 (Jackson ImmunoResearch Labs, West Grove, PA, USA). Embryos and tissues were imaged on a Leica SP8 confocal microscope (Leica Microsystems GmBH, Wetzlar, Germany) using a 40× immersion objective. Developmental Studies Hybridoma Bank (DSHB), developed under the auspices of the National Institute of Child Health and Human Development (NICHD) and maintained by the University of Iowa, Department of Biology. 

### 2.3. DNA Constructs and Generation of Transgenic Lines

The HeyUP^FL^ fragment (2669 nucleotides (nt)) was PCR amplified from fly genomic DNA (150 ng) using Taq Polymerase (Minotech Biotechnology) with the primers FORHeyup and REVHeyup (Table 2, I), bearing linkers with the EcoRI and Kpn I restriction sites (underlined letters), respectively. The PCR program used was as follows: (1) 94 °C for 5 min, (2) 94 °C for 30 s, (3) 55 °C for 30 s, (4) 72 °C for 2.5 min, (5) steps 2–4 30×, and (6) 72 °C for 5 min. The fragment was cloned into the EcoRI and KpnI sites of placZ.attB [35] (provided by Konrad Basler lab), and pnlsGFP.attB vectors, generating the HeyUP^FL^-nbgal and HeyUP^FL^-nGFP transgenes, respectively. The HeyUP^2+3SH^-nGFP transgene was generated by PCR amplification of the fragment HeyUP^2+3SH^ (2499 bp) using the primers heyPf-E1 and REVHeyup (Table 2, I) and subsequent cloning into the pnlsGFPattB vector. The HeyUP^NSH^ fragment (2275 bp) was PCR amplified using the primers UPMBF and REVHeyup (Table 2, I) and cloned into the HSB-GsF(pH Stinger attB) [36], giving the HeyUP^NSH^-nGFP transgene.

PCR amplification of the Hey intron 2 sequence (734 bp) was performed using fly genomic DNA(150 ng) with Taq Polymerase (MINOTECH Biotechnology) using the primers heyIf-X1 and heyIr-K1 (Table 2, I), bearing linkers with XhoI and Kpn I restriction sites (underlined letters), respectively. The PCR program used was as follows: (1) 94 °C for 2 min, (2) 94 °C for 1 min, (3) 45 °C for 1 min, (4) 72 °C for 2.5 min, (5) steps 2–4, 5×, (6) 94 °C for 1min, (7) 48 °C for 30 s, (8) 72 °C for 2.5 min, (9) steps 6–8 30×, and (10) 72 °C for 10 min. The fragment was initially cloned into the pSL1180 vector (Addgene, Watertown, MA), and from there it was subcloned as an XbaI fragment into the XbaI site of the placZ.attB and pnlsGFP.attB vectors to generate the HeyIN2-nbgal and HeyIN2-nGFP transgenes, respectively.

Mutagenesis of Su(H) binding sites of the HeyIN2 region: A 894 bp XhoI-BamHI fragment, including the HeyIN2 sequence, was isolated from the pSL1180-HeyIN2 plasmid, was subcloned into XhoI/BamHI sites of pBluescript SK and the construct was verified by sequencing (pBS-HeyIN2). For mutagenizing the Su(H) binding sites, primers (Table 2, II), including nucleotide substitutions (red letters) of the respective motifs (highlighted) which also generate new restriction sites, were used in PCR reactions with the pBSHeyIN2 plasmid as a template. Mutants were selected based on the presence of the relevant new enzyme restriction sites, and they were verified by sequencing. The HeyIN2^mSALL^ was then subcloned into the pnlsGFP.attB and placZ.attB vectors to generate the respective transgenes. 

All attB transgenes generated in this study were integrated into either the attP40 (2nd chromosome (chr)) or attP2 (3rd chr) landing sites using the phiC31 integrase system. Microinjections of plasmid DNA were carried out into *nanos- ΦC31*; *attP* fly embryos., Flies that grew to adulthood were crossed with *yw^67c23^* flies, and the mini-*white* marker was used for tracking the transgene in the subsequent screening.

### 2.4. Generation of Df(2R)Hey^7552/6656^ Mutant

**Figure 1:**
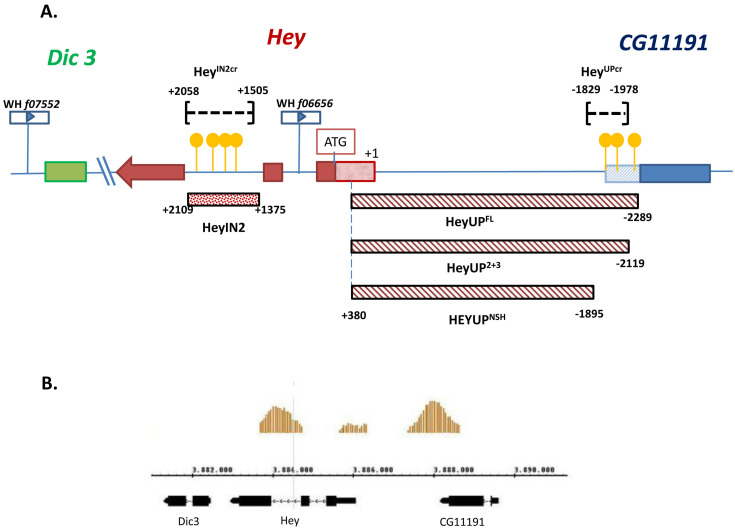
***Hey* locus and its neighboring CG11191 upstream and *Dic3* downstream genes.** (**A**) *Hey* exons are presented as red-colored boxes and 5′UTR (starting at +1) is presented as a light red-colored box. The CG11191 exon is indicated by a blue-colored box, with its 3′UTR designated as a light blue hatched box, and the *Dic3* locus is indicated by a green-colored box. Yellow spheres represent Su(H) binding sites. Dotted and hatched bars with coordinates at either end correspond to the DNA fragment of the second intron and the truncated fragments of the upstream region, respectively, which were used in the enhancer reporter transgenes as designated. Dashed lines within brackets represent the deletions, induced by CRISPR/Cas technology, that removed the Su(H) binding sites from the Hey intron 2 and upstream regions. The positions of the two *piggyBac* insertions WH(+)f07552 and WH(+)f06656 used for the generation of *Df(2R)Hey^7552/6656^* mutant are indicated downstream of *Dic3* and within the *Hey* first intron, respectively. (**B**) View from a genome browser (*Drosophila melanogaster* version dm3) encompassing the *Hey* gene. The Su(H) ChIP-on-chip signal from larval CNS chromatin [37] is plotted above the DNA map.

A deletion of the *Hey* gene coding sequence (CDS) was generated by the FRT-based deletion strategy [31], which is based on flippase-induced trans-recombination between FRT sites included in transposon insertions and subsequent PCR-based identification of the deletion event. The two *piggyBac* insertion lines used were *WH(+)f*07552 [44A2, 2R:7992094] and *WH(+)f06656* [44A2, 2R:7997576], isolated during the Exelixis screen project [32], each carrying a single transposon inserted either downstream of *Hey* [7995438…7998278] and its neighboring locus *Dic 3* (CG11196) [7993798…7994968] or in the first intron of *Hey*, respectively (Figure 1A). Both transposons contain an FRT site in the same orientation, a prerequisite for generating a FRT-based deletion. The position of both FRTs is 5′ relative to the *w*^+^ transgene of each transposon so after recombination the *w*^+^ marker is retained [31]. Since recombinants and non-recombinants are both w+, the visible marker mutations *al, dp^ov1^*, *px sp^1^* were used to discern the recombinants. The strains *al*, *dp^ov1^ WH(+)f*07552, and *WH(+)f06656 px sp^1^* were generated by standard meiotic recombination crosses and were then used in deletion generation crosses carried out according to the scheme described in [31]. Putative FRT-based deletion events were recovered over the chromosome bearing the *al*, *dp^ov1^*, *px sp^1^* mutations by selecting individuals from the progeny of the final cross that were homozygous for all four visible markers. The deletion event was subsequently confirmed by genomic two-sided PCR screening [31] of these individuals that detected the residual ends of both elements in the same individual. The element-specific primers Tn2FWD and Tn1REV [31], paired with the corresponding genome-specific primers GP6656REV and GR7552FWD (Table 2, III and Supplementary Figure S6A), were used to produce PCR fragments of 866 bp and 720 bp, respectively. Six out of ten selected individuals were positive for the deletion event based on the two-sided PCR screening, a gel image-example of which is presented in Supplementary Figure S6B. Finally, the deletion-bearing chromosomes were recovered over a twistG>UASGFP-marked *CyO* balancer chromosome to enable the selection of mutant embryos for viability assays.

For monitoring the embryo viability of Hey mutants, 3 h collections of GFP^-^ and GFP^+^ embryos corresponding to the homozygous mutant and the heterozygous mutant/CyO, twistG>UASGFP, respectively, were performed. Embryos were aged at 25 °C for 24 h to allow hatching, and dead embryos were counted. Six different experiments of 100–150 individuals were performed, and the percentage of viability was calculated for each one. For the statistical analysis, one-way ANOVA with Tukey’s post-test (comparison of all pairs of columns) was performed using GraphPad Prism version 5.00 for “Windows(GraphPad Software, San Diego, CA, USA, www.graphpad.com, accessed on 2 July 2024)”. 

### 2.5. CRISPR-Cas9 Induced Deletions in the Hey Locus

CRISPR/Cas9 genome editing technology was applied for deleting sequences from Hey upstream and Hey Intron2 genomic regions that include two and four Su(H) binding sites, respectively (Figure 1A). Firstly, a 646 bp HeyUP [A] and a 734 bp HeyIN2 [B] fragment were PCR amplified from the genomic DNA of the transgenic strain y^1^ M{nanos-Cas9.P}ZH-2A w* [33] using KAPA Hifi polymerase and the pairs of primers UPFip/UPRip [A] and heyIf-X1/heyIr-K1 [B] (Table 2, I). The PCR program used for [A] was as follows: (1) 95 °C for 5 min, (2) 98 °C for 20 s, (3) 65 °C for 15 s, (4) 72 °C for 15 s, (5) steps 2–4 30×, and (6) 72 °C for 5 min. For [B], the following PCR program was used: (1) 95 °C for 5 min, (2) 98 °C for 20 s, (3) 65 °C for 15 s, (4) 72 °C for 1 min, (5) steps 2–4 35×, and (6) 72 °C for 5 min.

The DNA sequence of the two fragments was determined and used in the flyCRISPR Optimal Target Finder tool http://tools.flycrispr.molbio.wisc.edu/targetFinder/ accessed on 2 July 2024) to find highly specific target sites for the design of the gRNAs (Table 2, IV and Supplementary Figure S7). All gRNAs were cloned into the BbsI site of the pU6-BbsI-chiRNA plasmid (Addgene) so that they were expressed under a U6 promoter [38]. A DNA mix of 100 ng/μL of each of the two vectors containing the relevant gRNAs either for HeyUP or HeyIN2 deletions was injected into embryos (0–3 h) of the transgenic strain y^1^ M{nos-Cas9.P}ZH-2A w*, which expresses Cas9 during oogenesis under the control of *nanos* regulatory sequences [33]. Hatched larvae were transferred in vials with fly food medium, and the flies that grew to adulthood were used in the following pipeline of genetic crosses and PCR screening detection of CRISPR events, in order to establish each one of the three CRISPR mutant lines of the study.

1. Adults surviving the injection were mated individually with *yw^67c23^*(wt) flies. DNA from a pool of 30 F1 progeny (flies and/or pupae) of each cross was extracted and was used in a PCR screen to look for the expected CRISPR deletion product (CR event) (**positive P cross**).

2. A total of 10-15 F1 progeny [*yw*;CR//+ or *yw*;+//+] from each of the positive P crosses were mated individually with Df(2R)*Hey*^7552/6656^[*w^+^*]//*CyOwglacZ(w^−^)* flies. DNA from each of the mated F1s was extracted after they had generated an adequate number of offspring (F2) and was used in the PCR screen for the CR event (**positive F1**).
3. Selection against the Df(2R)*Hey*^7552/6656^[*w^+^*] chromosome among the progeny of the positive F1s. Individual (*w^−^*) F2 progeny (*yw*; CR//*CyOwglacZ* or *yw*; +//CyOwglacZ) were mated with Df(2R)*Hey*^7552/6656^[*w^+^*]//*CyOwglacZ(w^−^)* flies. DNA from each of the mated F2s was extracted after they had generated an adequate number of offspring (F3) and was used in the PCR screen for the CR event (**positive F2**).
4. Selection against Df(2R)*Hey*^7552/6656^[*w^+^*] phenotype among the progeny of the positive F2s and inter-se cross of F3 *yw*; CR//*CyOwglacZ* virgin female and male siblings in order to establish the CRISPR deletion mutant line.

For HeyIN2 CRISPR deletion, embryo injections generated 48 fertile adults that entered the above pipeline. Three deletion events were initially identified, but only two of them were finally recovered, establishing two separate *Hey^IN2cr^* mutant lines. Accordingly, for HeyUP deletion, 31 fertile adults entered the pipeline, and 3 deletion events were finally recovered, establishing 3 different *Hey^UPcr^* mutant lines. For the generation of mutant lines carrying both deletions, the plasmids carrying the UP5′ and UP3′gRNAs were injected into embryos of the *Hey^IN2cr^* mutant line in which the X chromosome had been substituted by the X chromosome of the y^1^ M{nanos-Cas9.P}ZH-2A w* strain carrying the nanos-Cas9 transgene. The injections generated 40 fertile adults that were used in the pipeline scheme. At the end, four HeyUP CRISPR deletion events were recovered in the *Hey^IN2cr^* genetic background, and the relevant *Hey^UPcrIN2cr^* double mutant lines were established. 

Genomic DNA extraction from either 30 F1 flies/pupae or single fly progeny was performed using 500 μL or 50 μL DNAzol reagent (Invitrogen), respectively, and ~100 ng of it was used in the PCR reactions. The primers used flanked the deletion area and are described in Table 2, I. Gel images representative of the PCR screening of the parental crosses are presented in Supplementary Figure S8. Concerning the Hey upstream region, using the primers UPFip and UPRip, a 646 bp product is normally amplified unless a CRISPR deletion event is induced, producing a 497 bp fragment (Supplementary Figure S8A,D). The HeyIN2^cr^ deletion was identified using heyIf-X1 and heyIr-K1 primers at the 5′ end and the 3′ end of Hey intron2 that amplify either a 734 bp wild-type fragment or a 181 bp product representing a CRISPR event (Supplementary Figure S8B). After establishing the mutant lines, the IN2 deletion event was also verified by PCR amplification using a different pair of primers, namely CDSF (located at the beginning of *Hey* coding region) and heyIr-K1, which produce either a 1628 bp wild-type fragment or a 1075 bp deletion one, respectively (Supplementary Figure S8C).

## 3. Results

### 3.1. Identification of Two Enhancers from the Hey Locus

Previous work has shown that expression of *Hey* gene in the nervous system of *Drosophila* during embryonic and larval stages displays complex patterns that are either dependent or independent from Notch signaling [25]. As a first step towards understanding this dual mode of Hey expression, we searched the sequence of *Hey* genomic locus for the presence of a **YGTGRGAA** motif that is indicative of Notch-dependent regulation as it represents a high-affinity binding site consensus sequence of the Notch DNA tethering factor Su(H) [17,19]. Our search revealed three copies of this motif upstream of the *Hey* transcription unit at positions -2235nt (upS1), -1947nt (upS2), and -1922nt (upS3), which are included in the 3′UTR of the uncharacterised neighbouring gene CG11191. Four more copies of the Su(H) consensus motif were found within the second intron of the *Hey* gene at positions +1708 (inS1), +1808 (inS2), +1896 (inS3), and +2026 (inS4) (Figure 1A and Supplementary Figure S1). In support to this analysis, the above two regions were revealed by ChIP-on-chip experiments for genome-wide Su(H) binding [37] as presenting significant enrichment of the Su(H) ChIP signal (Figure 1B). Furthermore, we investigated the putative conservation of the particular sequence sites in the corresponding genomic regions of eight different *Drosophila* species (*melanogaster*, *simulans*, *sechellia*, *erecta*, *yakuba*, *ananassae*, *pseudoobscura*, and *persimilis*). EvoPrinter analysis [39] showed that all three Su(H) binding sites in the *Hey* upstream region (upS1, upS2, upS3) and three out of the four sites of the second intron (inS2, inS3, inS4) are conserved in all eight species (Supplementary Figure S1), while the inS1 site is conserved only in five of them (*melanogaster*, *simulans*, *sechellia*, *erecta*, and *yakuba*). In conclusion, the above analysis identified multiple conserved Su(H) binding sites both upstream of the *Hey* transcription unit and within the second intron of the locus, indicating that either or both of these regions may serve as putative regulatory elements of *Hey* expression in response to Notch signaling.

To investigate this possibility, we initially isolated fragments of genomic DNA corresponding to the two regions. A fragment of 2669 bp (HeyUP^FL^) that includes 374 bp of the CG11191 3′ UTR, 1915 bp of the intergenic sequence, and 380 bp of Hey 5′UTR (Figure 1A) and a 734 bp fragment (HeyIN2) including all four Su(H) binding sites of the second intron (Figure 1A) were PCR amplified and then used to drive the expression of nuclear β-galactosidase or nuclear GFP in vivo*,* in transgenic lines which bear the relevant transgenes inserted either in the attP40 (2nd chr) or the attP2 (3rd chr) sites. By performing an X-gal assay in third instar larval CNSs from the respective β-galactosidase lines (Supplementary Figure S2), we showed that both cloned regions drive b-galactosidase activity at about the same levels and in similar patterns resembling those of the *Hey* gene expression. 

In all the following experiments, we present a more detailed analysis of the expression pattern of the two reporters using immunohistochemistry, comparing them to endogenous Hey expression as detected by a Hey specific antibody [25] in the nervous tissue of embryos and larvae as well as in the developing midgut of the embryo.

### 3.2. Both HeyUP^FL^ and HeyIN2 Enhancers Are Active in the Embryonic CNS

Within CNS, Hey is firstly detected at early stage 11 in a segmentally repeated pattern of newborn neurons that is gradually enriched with more Hey-positive cells, being evident until the late embryonic stages. The expression of Hey characterizes primarily immature neurons and an uncharacterized subset of Repo-positive glia [25]. We examined the two enhancer elements as to their capacity to drive the expression of either b-galactosidase or GFP reporters in accordance with the expression pattern of Hey.

**Figure 2:**
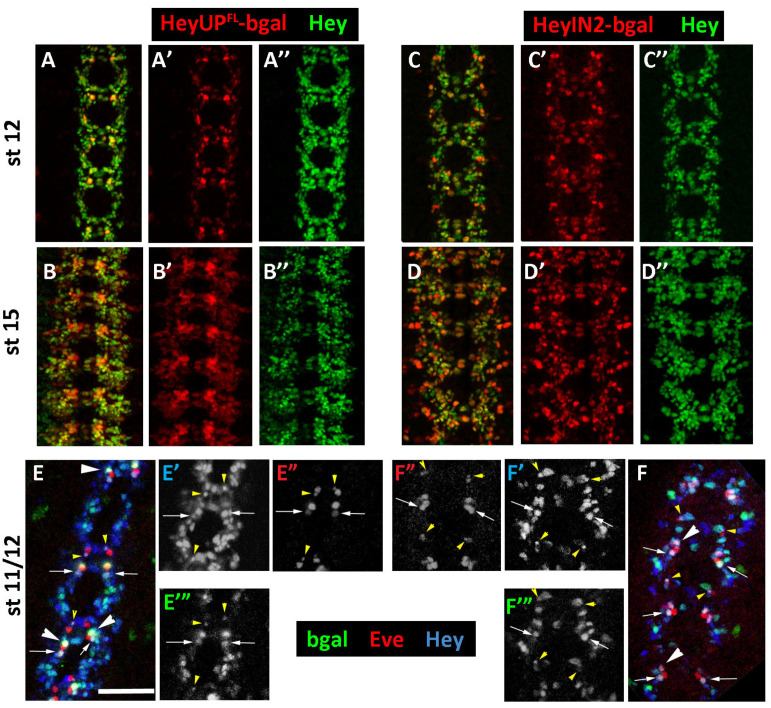
**Expression of Hey enhancers’ reporters in the embryonic CNS.** (**A**–**D”**): Ventral views of stage 12 and stage 15 embryos stained for β-galactosidase (red) and Hey (green); anterior is up. HeyUP^FL^-bgal expression in stage 12 embryo (**A**–**A”**) coincides with endogenous Hey but it is not turned on yet in all Hey-positive cells. Differential intense staining of b-gal is evident in a repeated pattern of 2-3 Hey-positive cells either side of the midline. At stage 15 (**B**–**B”**), all Hey-positive cells are HeyUP^FL^-bgal positive as well, and more b-gal intensely stained cells have been added in the medial clusters. (**C**–**D”**) Expression of HeyIN2-bgal recapitulates the expression pattern of Hey in early (st12, (**C**–**C”**)) and late (st15, (**D**–**D”**)) embryo stages. Few cells in the peripheral sites along the VNC exhibit intense b-gal staining in both stages. Scale bars: 50 μm (**A**,**C**), 46 μm (**B**), and 43 μm (**D**). (**E**–**F’”**): Hey reporters in Eve positive lineages. Stage 11/12 embryos showing deep focal planes with aCC/pCC and RP2/RP2sib pairs marked with Eve (red in (**E**,**F**)). HeyUP^FL^-bgal (**E**) and HeyIN2-bgal (**F**) in green are co-expressed with Hey (blue in (**E**,**F**)) in pCC (arrow) but not aCC (red cell next to arrow). Similarly, co-expression is evident in RP2sib (yellow arrowhead) which can be still distinguished at this stage as an Eve expressing cell, as well as in Hey/Eve positive U cells (large white arrowhead) evident in some segments. (**E**’–**E’”**) and (**F’**–**F’”**) images are single channels of E and F in black and white designated with colored letters corresponding to the respective channel. Scale bar: 40 μm.

Figure 2 displays the expression pattern of HeyUP^FL^-bgal and HeyIN2-bgal reporters (stage 11/12 and stage 15) which is evident from early stage 11 through stage 15–16. In general, the expression of both reporters closely follows endogenous Hey, although quantitative differences were noticed in distinct positive cell populations. Specifically, HeyUP^FL^-bgal (Figure 2A–B”) displays particularly strong expression in a group of Hey-positive cells [(2–3 cells as early as stage 11/12 (Figure 2A’) while later on a few more are added (Figure 2B’)] in every hemisegment and in either side of the midline, forming a characteristic segmented pattern along the VNC. The rest of the Hey-positive cells exhibit much lower levels of b-galactosidase, even undetectable in some of them at stage 11/12. This is attributed though to a delay in b-galactosidase accumulation, as at later stages all Hey-expressing cells have turned on the marker. On the other hand, the HeyINT2-bgal reporter (Figure 2C–D”) displays a more uniform expression in the majority of Hey-positive cells, although several of them, in peripheral sites along the VNC, express higher levels of the marker (Figure 2C’,D’). These correspond mostly to Hey-positive glia, confirmed by colocalization with the glia marker Repo (Supplementary Figure S3A’–A’”, arrowheads). In addition, several other Hey-negative glia are among the HeyIN2-bgal positive cells (Supplementary Figure S3A,A”, arrows), which we refer to as the ectopic expression of the reporter. A group of such HeyIN2-positive/Hey-negative cells belong to the characteristic class of longitudinal glia [40] that are positioned along the CNS dorsal midline (Supplementary Figure S3B–B’”, large arrows). In conclusion, despite the fact that HeyUP^FL^- and HeyIN2-driven reporters are strongly expressed preferentially in different subsets of Hey-positive cells, both enhancer elements seem to be active in an overlapping pattern that fully recapitulates that of the endogenous Hey, indicating that, during primary neurogenesis, Hey expression is potentially regulated by both of them. In addition to CNS, extensive expression of both reporters was also detected in the developing PNS, which was not studied further.

In an attempt to further analyze the activity pattern of the two regulatory units of the *Hey* locus and potentially uncover differences not easily detected in the bulk neuronal population, we examined the expression of our reporters in specific subsets of sibling neurons with unequal fates that differentially express Hey in response to Notch signaling [25]. We used the lineage-specific marker Even-skipped (Eve) that marks the aCC/pCC and RP2/RP2sib sibling pairs, the cluster of U motor neurons, and the cluster of EL interneurons. By using this marker, we have previously shown that Hey is expressed in the pCC, RP2sib, and U neurons, transiently during their birth and in response to differential activation of Notch signaling in them. In agreement with that, both HeyUP^FL^-bgal and HeyIN2-bgal reporters are expressed in the same sibling neurons as Hey (Figure 2E,E’,F,F’, respectively). It is also worth mentioning here that, in the process, we noted that a subset of the neurons close to the midline that strongly express HeyUP^FL^-bgal (Figure 2E,E’) correspond to the U neurons.

### 3.3. HeyUP^FL^ and HeyIN2 Enhancers Support Hey Expression in the Developing Midgut

**Figure 3:**
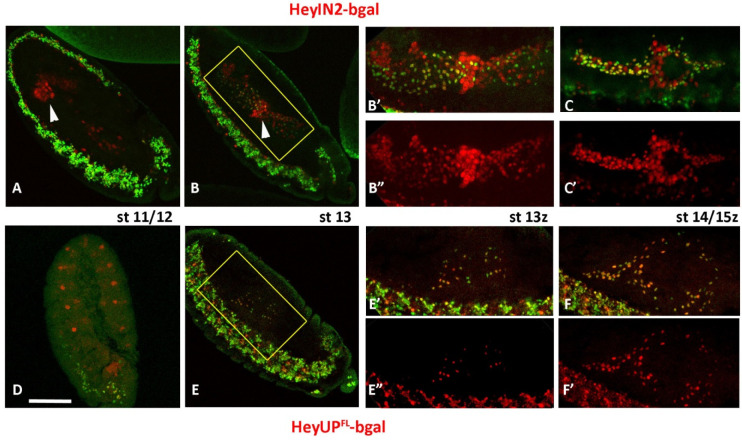
**Hey enhancers in the embryonic midgut primordia.** Sagittal views of different stage embryos, as designated, that are stained for Hey (green) and b-galactosidase (red). Anterior is right and ventral is down. (**A**) In stage 11 germ band-extended embryos, ectopic expression of HeyIN2-bgal is detected in a few large nuclei in the pmg (arrowhead). (**B**–**B”**) In the stage 13 embryo, amg and pmg primordia have fused and the ectopic HeyIN2-bgal positive large nuclei (white arrow) are detected at the fusion point. From this stage on, expression of HeyIN2-bgal is evident in the Hey-positive EE cells ((**B’**–**B”**), st13 and (**C**–**C’**), st14/15). (**D**) Stage 11 embryo showing HeyUP^FL^-bgal ectopic expression in presumptive PNS primordia. (**E**–**F”**) HeyUP^FL^-bgal expression is evident in Hey-positive EE cells initially at stage 13 (**E**–**E”**) and in later stages (**F**–**F’**). Scale bars: 80 μm (for **A**,**B**,**D**,**E**); 55 μm (for the rest).

In the developing midgut primordia of stage 12/13 embryos, enteroendocrine precursors (pre-EE) divide once to generate two Prospero-positive enteroendocrine cells of different fates upon differential Notch signaling [41]. We have previously shown that Hey is expressed after stage 13 in almost half of the Prospero-positive EE population, and that its expression there is Notch-dependent [27]. In accordance with that, both HeyIN2-bgal and HeyUP^FL^-bgal reporters co-express with endogenous Hey in the same EE subpopulation at stage 13 (Figure 3B–B”,E–E”, respectively) as well as in later stages (Figure 3C,C’ and Figure 3F,F’, respectively). In stage 13, some of the Hey-positive cells do not have detectable levels of the b-gal marker of either reporter which is attributed to a delay in b-galactosidase accumulation, as later on the marker is turned on in all Hey-expressing cells. Additionally, the expression of both reporters was observed as early as stage 11/12, which we refer to as ectopic expression. The HeyUP^FL^ enhancer that seems to fully recapitulate the Hey expression in the developing midgut (Figure 3E–F’) is ectopically active in a segmented pattern of ectodermal cell clusters that resemble the pro-neural clusters of the PNS (Figure 3D). On the other hand, ectopic expression of HeyIN2-bgal reporter was observed, in large nuclei in front of the migrating posterior midgut primordia (pmg) at stage 11 (Figure 3A, arrowhead) and even earlier. At stage 13, these nuclei are positioned at the site where the anterior and posterior primordia fuse (Figure 3B–B”, arrowhead), while later on (stage 15), they form a stripe around the middle of the midgut sac (Figure 3C,C’). Based on their characteristic size and location, we assume that they correspond to the interstitial cell precursors (ICPs) [41,42] that give rise to the interstitial/copper cells, a group of characteristic enterocytes of the acidic middle region of larva midgut [43,44]. In addition to presumptive ICPs, ectopic expression of HeyIN2-bgal reporter was also observed in a few scattered Hey-negative nuclei along the developing midgut both in early and late stages (Figure 3A–C’), the identity of which was not investigated further. In conclusion, the HeyUP^FL^ enhancer seems to be more representative of Hey expression in the embryonic midgut, while HeyIN2, though recapitulating Hey expression in the EE cells, is active in other midgut cell populations as well.

### 3.4. HeyUP^FL^ and HeyIN2 Enhancers Are Active in Larval CNS

Previous mosaic analysis in larval CNS has suggested that the expression of Hey in the majority of postembryonic neuronal lineages of the central brain, VNC, and optic lobe is dependent on Notch signaling via the canonical Su(H)-dependent pathway. We examined the expression pattern of the HeyUP^FL^-GFP and HeyIN2-GFP reporters in the larval CNS in comparison to the endogenous Hey expression. During the second and third larval stages, many embryonic NBs that have ceased their divisions at the end of embryogenesis resume asymmetric divisions, generating a second burst of proliferation. As Hey embryonic expression is transient, it is no longer detectable in the primary neurons even in young larvae; thus, accumulation of Hey in larval CNS is detected only in groups of immature secondary neurons positioned near the surface of the CNS, next to each NB of the central brain and the thoracic ganglia of the ventral nerve cord (VNC). Additionally, Hey is also detected in immature neurons within both proliferation centers of the optic lobes, in a few cells in the abdominal ganglion, as well as in several Repo-positive glia in optic lobes and the central brain [25].

**Figure 4:**
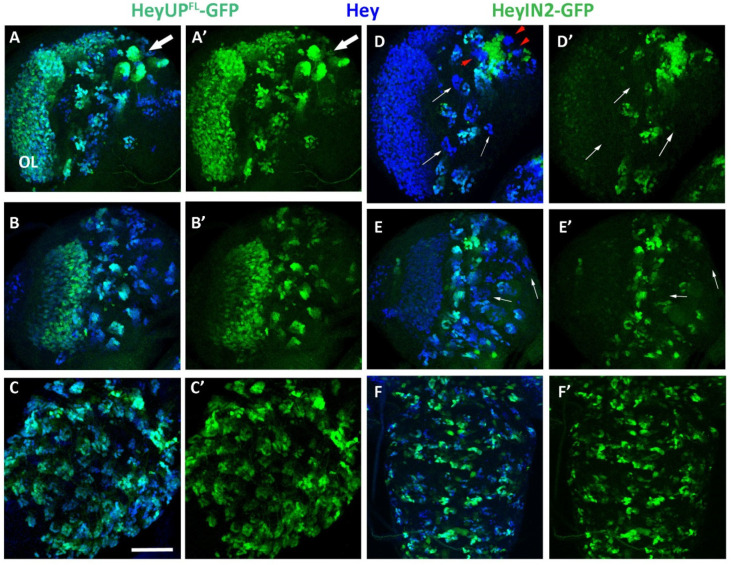
**Expression of Hey enhancers’ reporters in larva CNS**. Projections of confocal optical sections showing third instar larval brain hemispheres and VNC. Anterior is top, lateral is left. (**A**–**C’**) HeyUP^FL^-GFP (green) overlaps with Hey (blue) in all lineages of the central brain, with dorsal (**A**–**A’**) and ventral (**B**–**B’**) focal plains, as well as in the thoracic ganglia of the VNC (**C**–**C’**). Arrow points to 3 MB lineages in which Hey-positive Kenyon cells are also positive for HeyUP^FL^-GFP. (**D**–**F’**) HeyIN2-GFP (green) overlaps with Hey (blue) in the majority of central brain lineages and in all lineages of VNC thoracic ganglia. Small white arrows point to lineages located in the dorsal (**D**–**D’**) and ventral (**E**–**E’**) focal planes of brain hemispheres in which the enhancer is not active. Red arrowheads in (**D**) point to 3 MB lineages where HeyIN2 enhancer is not active in the Hey-positive cells. Note that the enhancer is turned on in the older population of Kenyon cells next to the Hey-positive young ones. Scale bars: 48 μm (**A**,**B**,**D**,**E**), 40 μm (**C**), and 60 μm (**F**).

HeyUP^FL^-GFP expression recapitulates the endogenous Hey pattern (Figure 4A–C’). Hey-expressing cells of all central brain lineages, on the dorsal (Figure 4A,A’) and ventral side (Figure 4B,B’) and the thoracic ganglia of the VNC (Figure 4C,C’) are labeled by the GFP marker. It is noteworthy that GFP expression levels differ among the lineages, with some displaying higher levels than others, and with newborn Kenyon cells located next to the four NBs of the MB lineages exhibiting the highest levels (arrow in Figure 4A,A’). Strong colocalization of GFP and Hey expression is also observed in the outer (OPC) and inner (IPC) proliferation centers of the optic ganglia (Figure 4A–B’ and Figure S4D’,D”). HeyIN2 enhancer is also active in Hey-expressing cells in the central brain, VNC, and the optic ganglia (Figure 4D–F’ and Figure S4D–D’”), as well as in a few Hey-positive cells (glia and neurons) in the midline of the VNC. However, activation of HeyIN2 enhancer does not absolutely mirror the endogenous Hey expression. Although HeyIN2-GFP and Hey colocalize in the majority of Hey-positive lineages, a few lineages in the central brain are devoid of GFP expression (arrows in Figure 4D,D’,E,E’ and Figure S4A,A’, white arrows). Two are evident in ventral focal planes and three more are evident in the dorsal side, with two next to the optic lobe and one in a medial position. These are lineages where Hey expression may be driven exclusively by the HeyUP^FL^ enhancer, which is active there (Supplementary Figure S4A–A’ white arrows).

The most characteristic case of reporters’ differential expression is that of the four MB lineages, where we have previously shown that Hey expression is independent of Notch signaling [25]. Although newborn Kenyon cells are co-expressing Hey and HeyUP^FL^-GFP (described above), they are devoid of HeyIN2-GFP/bgal staining (Figure 4D, red arrowheads, Supplementary Figure S4B’). Interestingly though, HeyIN2 enhancer is ectopically active in Hey-negative older Kenyon cells next to the Hey-positive ones (Figure 4D,D’ and Figure S4B,B’). The above results indicate that the HeyUP^FL^ region includes the regulatory elements that control the Notch-independent expression of Hey within MB lineages. Activation of the HeyIN2 enhancer in the older Kenyon cells is negatively regulated when the enhancer is not in isolation but rather included in the Hey genomic locus. In addition to MB lineages, we observed two more instances of HeyIN2 ectopic expression. One was a crescent of Hey-negative cells between the two proliferation centers in the optic lobes (Supplementary Figure S4D–D’”). The second instance is numerous cells, not identified further, that are located mostly in deep layers of the central brain (Supplementary Figure S4D–D”’, white arrows) and in the abdominal ganglia. Therefore, *Hey* has a regulatory region that can drive its expression in additional cell types of the larval nervous system, where Hey protein is not normally detected (see Discussion).

### 3.5. Notch-Dependence of HeyUP^FL^ and HeyIN2 Regulatory Elements

In the light of two functional Hey enhancer elements, both of which include Su(H) binding sites, we wanted to address their dependence on Notch signaling and further investigate the Notch-dependent/independent regulation of *Hey* in the cells/tissues where it is expressed.

#### 3.5.1. Notch Deficient Background

**Figure 5:**
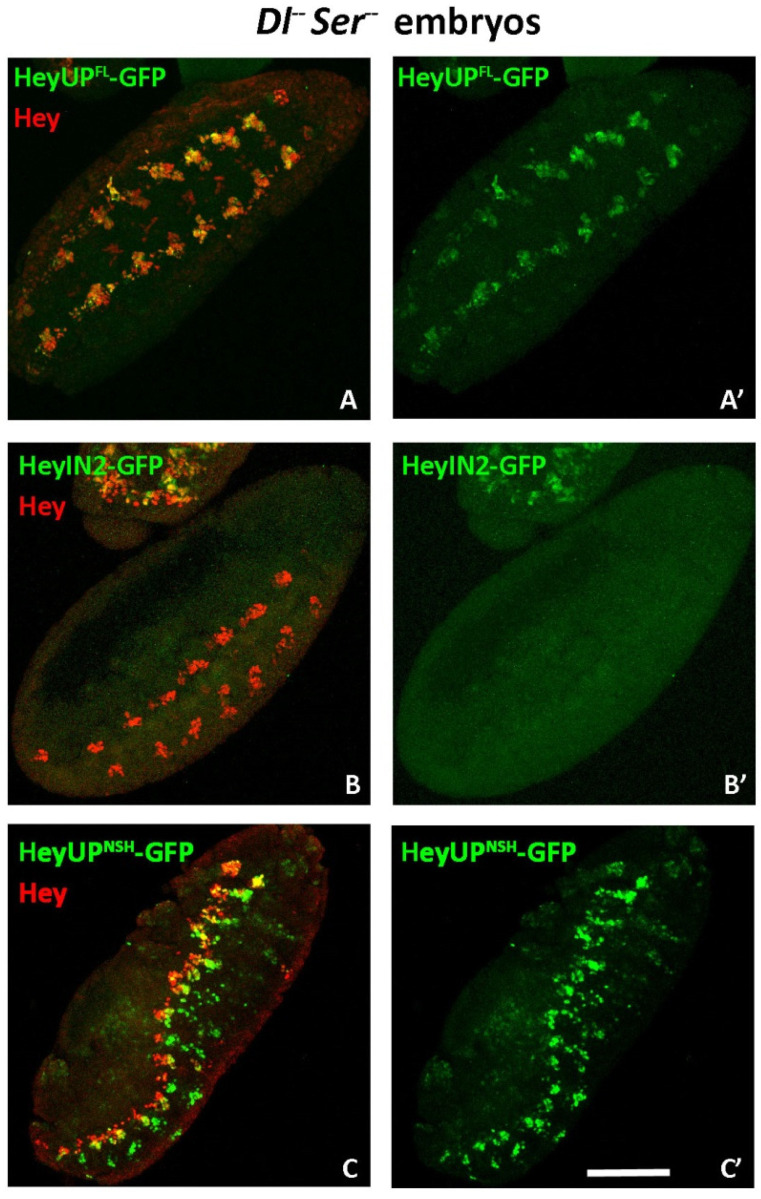
**Expression of Hey enhancers in a Notch-deficient background.** Semi-sagittal ventral views of *Dl^rev10^ Ser^RX106^* mutant embryos bearing HeyUP^FL^-GFP, HeyIN2-GFP, or HeyUP^NSH^-GFP transgenes stained for Hey (red) and GFP (green). Only a small number of cells in the *Dl^rev10^ Ser^RX106^* mutant CNS express Hey, and they are arranged in segmental groups forming bilateral rows along the VNC. These cells are also HeyUP^FL^-GFP-positive (**A**–**A’**), which suggests that Notch-independent expression of Hey is mediated by this enhancer. HeyIN2-GFP is not detected in the mutant CNS (**B**–**B’**) indicating absolute dependence of enhancer’s activation by Notch signaling. (**C**–**C’**) HeyUP^NSH^-GFP is sparsely detected in the Hey-positive clusters that persist in the *Dl^rev10^ Ser^RX106^* mutant CNS while the clusters of medial cells where it is ectopically expressed are enlarged, due to the neural hyperplasia of the *Dl^−^ Ser^−^.*deficient embryos. Anterior is up. Scale bar: 80 μm.

As a first step, we studied the expression of reporters driven by either HeyUP^FL^ or HeyIN2 regulatory regions in a genetic background deficient for *Delta* and *Serrate*, the two ligands of Notch, in which Notch signaling is completely abolished. The absence of Notch signaling in *Dl^-^ Ser^-^* mutant embryos results in a hyperplastic malformed CNS with an increased number of neuronal cells. Hey expression is evident in very few neurons, displaying a segmented pattern ([25] and Figure 5), and it has been attributed to a Notch-independent mode of *Hey* gene regulation. Interestingly, when monitoring HeyUP^FL^-GFP reporter in the mutant background, we observed an expression pattern that completely coincides with that residual Hey expression (Figure 5A,A’), On the other hand, HeyIN2-GFP reporter expression is completely absent (Figure 5B,B’). This result strongly indicates that during primary neurogenesis, Notch-dependent expression of Hey relies potentially on both HeyUP^FL^ and HeyIN2 regulatory units, while the Notch-independent mode is supported exclusively by HeyUP. Moreover, MARCM clonal analysis in the larva CNS, revealed that, similarly to Hey protein, expression of the two bgal reporters is abolished in double-mutant *Dl^−^ Ser^−^* clones (Supplementary Figure S5), further supporting the notion that Notch-dependent Hey expression can also be activated by either enhancer element in postembryonic neurons.

#### 3.5.2. Deletion/Mutational Analysis

In order to complement the above results, we applied different types of mutations targeting the Su(H) binding sites within each CRM, and using reporter transgenes we validated their impact in the enhancer functionality in the embryonic CNS and midgut, as well in the larval CNS. 

**Figure 6:**
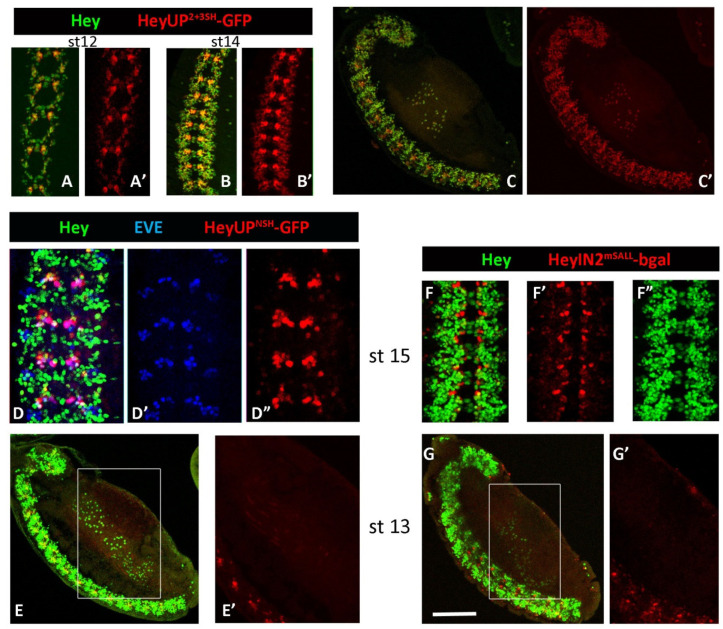
**Activity of the mutated Hey enhancers in embryo CNS and midgut primordia.** (**A**–**C’**) HeyUP^2+3SH^-GFP (red) expression in the stage 12 (**A**–**A’**) and stage 14 (**B**–**B’**) embryo CNS overlaps with Hey (green). Note the elevated intensity of the GFP staining in the area of U lineages in every segment compared with the rest of the GFP-positive cells. The low-ukp[omagnification image (**C**–**C’**) is the sagittal view of a stage 13 embryo and the projection of deep sections showing the developing midgut primordia. At this stage, expression of Hey is evident in a subpopulation of EE cells, and the same cells are co-expressing HeyUP^2+3SH^-GFP (**C’**). Scale bars: 62 μm (**A**); 90 μm (**B**,**C**). (**D**–**E’**) HeyUP^NSH^-GFP (red) expression is visualized.in the CNS of a stage 15 embryo (**D**–**D”**), in a subpopulation of Hey-positive cells (green). The majority of them form a segmented pattern (**D”**) that includes a U lineage in every hemisegment, as evidenced by the co-expression of the Eve marker (blue in (**D’**)) in several of them. The low-magnification image (**E**) is the sagittal view of a stage 13 embryo and the projection of deep sections showing the developing midgut primordia. HeyUP^NSH^-GFP (red) has been lost from Hey-positive (green) EE cells, as shown with the single channel in (**E’**) [higher magnification of the boxed area in (**E**)]. Only faint ectopic GFP staining is observed in unidentified cells. Scale bars: 32 μm (**D**), 90 (**E**), and 60 (**E’**). (**F**–**G’**) HeyIN2^mSALL^-bgal expression in stage 15 embryo CNS (**F**–**F”**). High expression of b-gal reporter (red, (**F’**)) is evident only in a few cells compared to the Hey (green, (**F”**))-positive population. Some of them are Hey-positive, but the majority are Hey-negative. Very faint b-gal staining is also observed in a few more cells along the VNC. Low magnification image (**G**) presents the sagittal view of a stage 13 embryo and the projection of deep sections showing the developing midgut primordia. Hey expression (green) is shown in the EE cell population, but HeyIN2^mSALL^-bgal (red) is not expressed at all, as shown in (**G’**) [higher magnification of the boxed area in (**G**)]. Scale bars: 45 μm (**F**), 90 μm (**G**), and 60 μm (**G’**).

**Figure 7:**
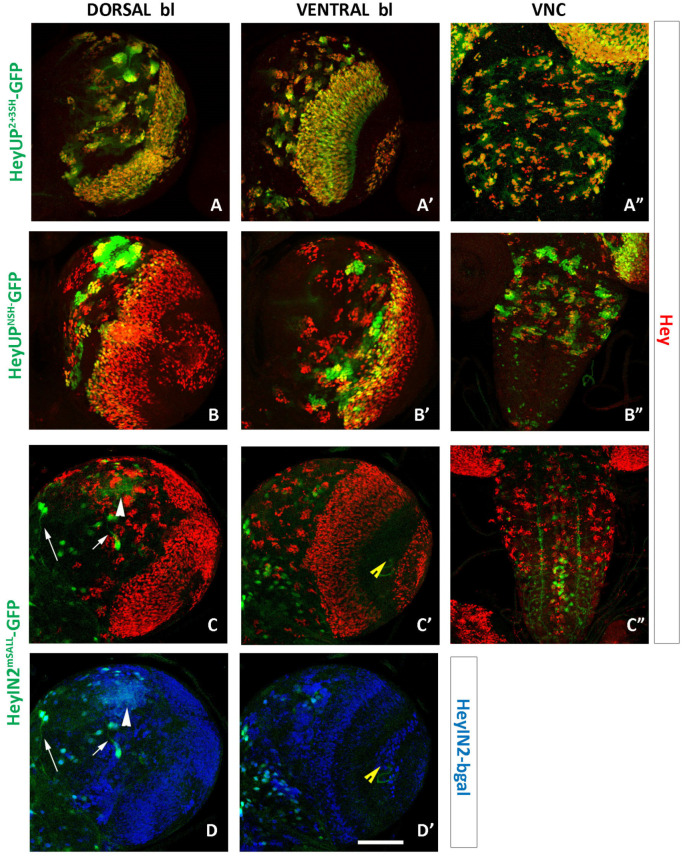
**Expression of mutated Hey enhancers’ reporters in larva CNS.** Projections of confocal optical sections showing third instar larval brain hemispheres and VNC. Anterior is top, lateral is right. (**A**–**A”**) HeyUP^2+3SH^-GFP (green) overlaps with Hey (red) in all lineages of the central brain (**A**,**A’**) and VNC (**A”**), similarly to HeyUP^FL^, as well as in optic ganglia cells. (**B**–**B”**) HeyUP^NSH^-GFP expression (green) is detected in Hey-positive cells of MB lineages as well as a few lineages in the dorsal (**B**) and ventral (**B’**) focal planes of the brain hemispheres. GFP perdurance is also visualized in older Hey-negative cells within these lineages. A few of the Hey-positive cells in the OL activate the enhancer as well. In the VNC (**B”**) several Hey-positive lineages in the thoracic ganglia and some scattered Hey-negative cells in the abdominal part are also positive for HeyUP^NSH^-GFP. (**C**–**D’**): HeyIN2^mSALL^-GFP (green) shows no overlap with Hey (red) or HeyIN2-bgal (blue) in larva CNS lineages. The mutant enhancer retains its activity only in ectopic sites of HeyIN2, namely old Kenyon cells of MB lineages (arrowhead) and old neurons deep in the brain hemispheres (arrows) (**C**,**D**), with a few in the thoracic neuromeres and a lot in the abdominal ganglia of the VNC (**C”**). In contrast, no GFP is detected in the ectopic site of HeyIN2 in the optic ganglia (yellow arrowhead in (**C’**,**D’**)). Scale bars: 67 μm, except for **B”** and (**C”**) (90 μm).

Two shorter fragments of the Hey upstream region were subcloned in transgenic vectors bearing the GFP reporter (see Materials and Methods). The HeyUP^2+3SH^ fragment (2499 bp) includes the two proximal Su(H) binding sites while HeyUP^NSH^ (2275 bp) does not include any of the three sites identified within the HeyUP^FL^ region. The expression of the HeyUP^2+3SH^-GFP reporter initiates as early as stage 11 and it is evident till late embryonic stages following the expression of endogenous Hey (Figure 6A–B’). More importantly, HeyUP^2+3SH^-GFP expression displays the same pattern and mode of differential intensity in CNS cell populations as the HeyUP ^FL^ reporter, and it is also observed in the Hey-positive cells of the developing midgut (Figure 6C,C’). In addition, identical expression pattern of HeyUP^2+3SH^- and HeyUP ^FL^-driven GFP reporters were observed in the larval CNS concerning all Hey-positive lineages in the central brain (Figure 7A,A’), VNC (Figure 7A”), and Hey-positive neurons of the optic ganglia. As the expression pattern of these two reporters is identical at all sites of Hey expression, we conclude that the contribution of the most distal Su(H) binding site is probably minimal and rather dispensable.

Expression of HeyUP^NSH^ GFP reporter in the embryonic CNS is firstly detected somewhat later (stage 12/13) than the onset of Hey expression (early stage 11) and it exhibits a very restricted pattern compared to HeyUP^FL^ or HeyUP^2+3SH^ reporters even in late embryo stages (Figure 6D”). It is quite interesting though that this pattern does not recapitulate the Notch-independent expression of Hey, as it is absent from the lateral clusters of Hey-positive cells that persist in Notch pathway mutants (Figure 5C). The above observation suggests that HeyUP^FL^ enhancer potentially includes a regulatory module upstream of the HeyUP^NSH^ fragment which drives expression in those cells. On the other hand, it is quite unexpected that the spatially restricted expression of the HeyUP^NSH^ reporter is primarily detected in medial cell groups overlapping with the cluster of U motorneurons in every segment as confirmed by colocalization with the Eve marker in both early and later stages (Figure 6D–D”). Upon closer examination, the HeyUP^NSH^-GFP-positive cells were often seen as doublets of a Hey-positive and a Hey-negative cell, suggesting that in these locations, expression may be activated in both siblings of an asymmetric division. The expression in both Notch-on and Notch-off cells was confirmed by examining HeyUP^NSH^-GFP expression in a *Dl^−^ Ser^−^* mutant background (Figure 5C,C’). The clusters of medial cells were enlarged, due to the neural hyperplasia of the *Dl^−^ Ser^−^* deficient embryos; these should contain Usibs and other Notch-off newborn neurons/glia (but not the U-motor neurons, which are absent in *Dl^−^ Ser^−^* mutant embryos [23]). At the same time, the HeyUP^FL^-mediated Notch-independent Hey expression in lateral clusters persists in the *Dl^−^ Ser^−^* mutants but is only sparsely accompanied by HeyUP^NSH^-GFP expression, because, as suggested above, the module responsible for this activity must have been deleted in the HeyUP^NSH^ reporter. Furthermore, concerning the developing midgut primordia, HeyUP^NSH^-GFP expression is absent from the Hey-positive EE cells (Figure 6E,E’) but rather a low staining is detected ectopically in unidentified cells (putatively muscles). Finally, in larval CNS, we observed HeyUP^NSH^-GFP expression in the Hey-positive cells of the 4 MB lineages as well as in a few more lineages in the dorsal and ventral sides of the central brain (Figure 7B,B’). Interestingly, in these lineages both Hey-positive and Hey-negative neurons express the HeyUP^NSH^ reporter. On the other hand, in the optic lobes, most Hey-expressing cells are devoid of GFP. In the VNC, three lateral and three medial Hey-positive lineages per hemi-neuromere of the thoracic ganglia as well as two more medial anterior ones in the first thoracic ganglion are GFP-positive (Figure 7B”). The above observations suggest that a large part of HeyUP^NSH^ expression in both embryos and larvae is ectopic, perhaps due to the loss of a repressive input. In some cases, this de-repression coincides with Hey-positive cells; in most cases, though, it happens in Hey-negative ones.

Lack of HeyIN2-bgal reporter expression in *Dl^−^ Ser^−^* mutant embryos suggests that point mutations induced by site-directed mutagenesis (see Methods), which destroy all four predicted Su(H) binding sites of Intron2 region (HeyIN2^mSALL^), should result in an inactive enhancer. Indeed, no expression of the HeyIN2^mSALL^-GFP reporter was observed either in embryonic Hey-expressing cells (CNS, gut) (Figure 6F–G’) or in Hey-positive lineages of larva CNS (Figure 7C–C”). Concerning the ectopic activation of the HeyIN2 enhancer, this is retained by HeyIN2^mSALL^ in presumptive glia cells of the embryonic CNS (Figure 6F,F’) as well as in the older Kenyon cells of MB lineages (Figure 7C,D, arrowhead) and in the cells within deep layers of the brain lobes and the VNC dorsal midline of larva CNS (Figure 7C–C”,D,D’). Interestingly, HeyIN2^mSALL^-GFP reporter expression in those cells identifies them as mature neurons, since residual cytoplasmic GFP staining nicely reveals their neuronal processes (Figure 7C” and arrows in Figure 7C,D). On the other hand, unlike wild-type HeyIN2, the mutated HeyIN2^mSALL^ enhancer does not drive GFP reporter expression in the large nuclei (presumptive ICPs) of the embryonic midgut primordia (Figure 6G,G’) and in the unidentified cells of the optic ganglia of the larva CNS (yellow arrowhead in Figure 7C’,D’), suggesting that HeyIN2 ectopic activation in these two sites requires Notch signaling, yet this Notch-dependent activation is suppressed when the enhancer is in its native state within the *Hey* locus. 

Combined, all the above results indicate that both HeyUP^FL^ and HeyIN2 enhancers have canonical Su(H)-dependent functions. HeyIN2 is activated mostly by Notch signaling during embryonic and larval life (even in its atypical expression domains, the large gut nuclei or the optic ganglia unidentified cells) displaying, however, a few Notch- and Su(H)-independent ectopic sites of expression (embryonic glia, old Kenyon cells, and neurons in deep layers of larva brain). On the other hand, the HeyUP^FL^ region supports N-dependent as well as N-independent expression of Hey. In particular, we identified two subregions that support the N-independent expression of Hey, one in the proximal promoter specific for MB Hey expression and the other further upstream. 

### 3.6. Functional Analysis of Hey Gene Using Genome Engineering

#### 3.6.1. Generation of a Null Hey Allele

The *Hey^f06656^* mutant refers to a piggyback recessive lethal insertion *WH-f06656* isolated in the first intron of *Hey* during the Exelixis screen [32] which results in undetectable levels of Hey protein in homozygous embryos [25]. Despite its lethality, this allele does not exhibit any apparent defects concerning the integrity of embryonic CNS [25]. This suggests either a probable redundancy between Hey and unidentified factor(s) or perhaps some residual activity of the transposon insertion providing partial cell specification activity at protein levels below the level of detection. 

In order to distinguish between the two scenarios, we generated a *Hey* null allele by applying the FLP/FRT recombination strategy described by Parks et al. (2004) [31] to induce a deletion with molecularly defined endpoints that removes a large portion of the *Hey* genomic locus. We took advantage of two piggyback insertion lines, *WH(+)f*07552 and *WH(+)f06656*, isolated during the Exelixis screen [32], which contain single FRT sites in the same orientation; thus, trans-recombination between them in the presence of FLP recombinase would generate a deletion tagged by residual element ends. As mentioned above, insertion *WH(+)-f06656* maps in the first intron of *Hey*, while in the line *WH(+)f*07552 [7992094…7992217], the insertion is 3.3 kb downstream of *Hey*, beyond the neighboring locus *Dic 3* (CG11196) [7993798…7994968] and a newly annotated lncRNA CR44272 [7992196…7992567] (Figure 1A and Figure S6A). We followed a scheme of deletion generation crosses as described in [31] and in the Materials and Methods section, and we identified the deletion by two-sided PCR screening of the progeny to detect residual element ends [31] (Supplementary Figure S6B).

We isolated several *Df(2R)Hey^7552/6656^* lines and verified them by the absence of Hey protein in homozygous embryos (not shown). The deletion mutant is embryonic lethal, and the lethality phase was determined in the embryo–larva transition. We concluded that this phenotype is due to the deficiency of the *Hey* locus and not of *Dic3* for the following reasons: (a) the Dic3 product is a dicarboxylate carrier of the mitochondria membrane which is expressed only in the pupa and presumably not required during embryonic stages. Additionally, a CRIMIC insertion [45] associated *Dic3* mutation results in a viable line (BDSC, BUN#94456). (b) The lethal phenotype resembles that of the *Hey^f06656^* insertion mutant. Typically, ~90% of mutant individuals die as late embryos and the remainder die as first instar larvae soon after hatching. (c) The same lethal phenotype is observed in *Df(2R)Hey^7552/6656^*//*Hey^f06656^* heterozygotes, where *Hey^f006656^* complements the *Dic3* and CR44272 loci. In conclusion, the phenotypic analysis of the two different *Hey* mutant alleles clearly shows that *Hey^f06656^* does not have residual activity and that *Hey* knockout indeed interferes with late embryonic life, allowing a few escaperes, which die as early larvae. 

#### 3.6.2. Generation of Lines with Deletions of the Su(H) Binding Sites from Hey Enhancers

Our data until now suggest that both HeyUP^FL^ and HeyIN2 regions activate *Hey* expression in response to Notch whereas HeyUP^FL^ additionally promotes the Notch-independent expression of *Hey*. In order to address whether the Notch-dependent component is redundantly mediated by the two enhancers and to deconvolve the impact of Notch-dependent vs. Notch-independent expression of the gene in the viability of the organism, we generated mutant *Drosophila* lines in which small deletions, engineered by CRISPR/Cas9 technology, eliminate the Su(H) binding sites from the *Hey* locus. Two different lines were initially produced, one bearing a 149 bp deletion around upS2 and upS3 of the *Hey* upstream region (*Hey^UPcr^*) and a second one with a deletion of 553 bp that removes all 4 Su(H) binding sites from the second intron of *Hey* gene (*Hey^IN2cr^*) (Figure 1A and Figure S7). In order to avoid sequence mismatches of the guide RNAs to the target sequences, we first PCR amplified and sequenced fragments containing the regions to be deleted from the genomic DNA of the transgenic strain y^1^ M{nos-Cas9.P}ZH-2A w** [33] that was used for the generation of our lines, and we designed the relevant guide RNAs (Supplementary Figure S7) based on the DNA sequence of these fragments. The deletion events were identified by a genetics/PCR screening protocol that also involved the *Df(2R)Hey^7552/6656^* mutant allele, according to the pipeline described in the Materials and Methods section. The primers used are described in Table 1, and the amplified fragments produced by a CRISPR-induced deletion event are either 497 bp (instead of 646 bp) for the upstream region or 181 bp (instead of 734 bp) for intron 2. Supplementary Figure S8 presents an example of gel images of the PCR screening process. 

**Figure 8:**
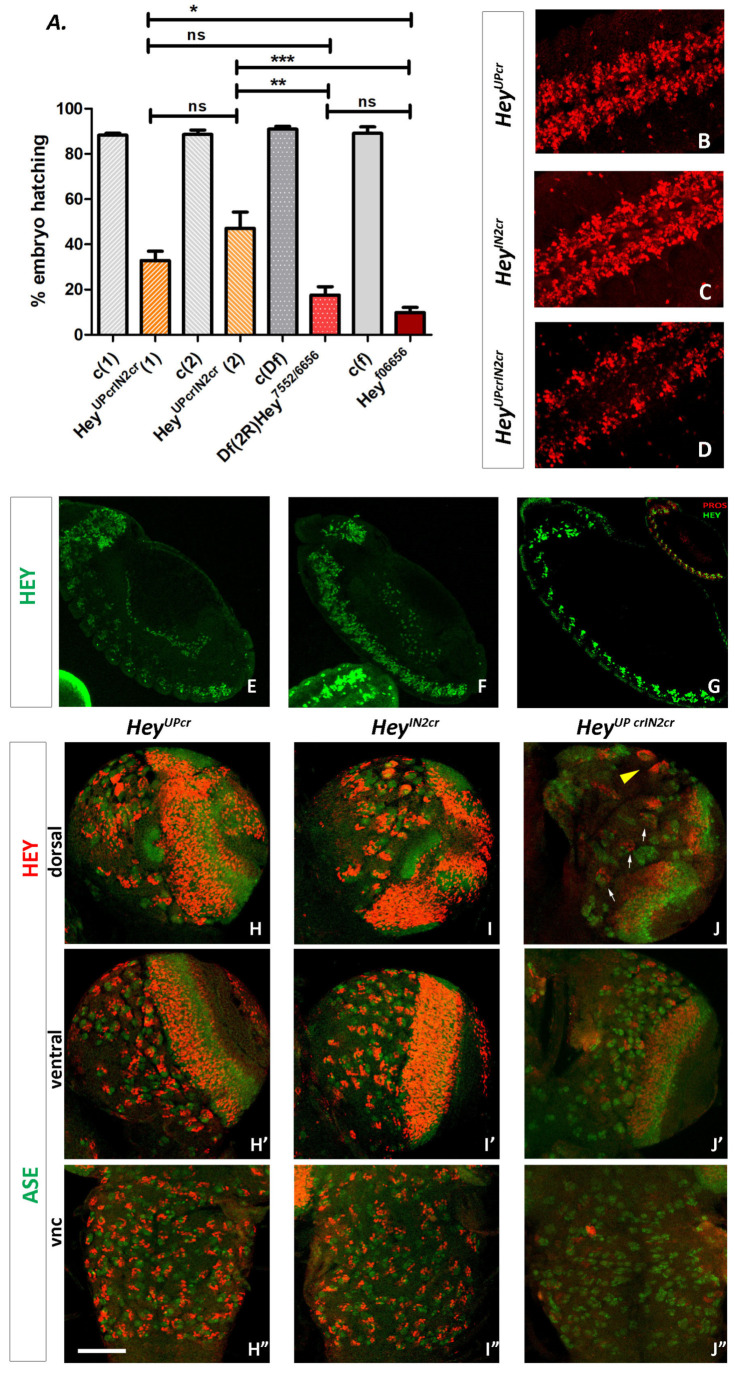
**Analysis of CRISPR *Hey* mutants.** (**A**) Diagram depicting the proportion of hatched embryos of indicated genotypes (GFP^-^: homozygous mutant genotype) and their internal controls (**C**) (GFP^+^: mutant genotype//CyO twiG>UGFP). Mean values with SEM from 6 different experiments of 100-150 individuals each. ns: non-significant, * *p* < 0.05, ** *p* < 0.01, *** *p* < 0.0001 (ordinary one-way ANOVA, Tukey’s multiple comparisons test). (**B**–**D**) Hey expression (red) in stage 15 embryos of *Hey^UPcr^* (**B**), *Hey^IN2cr^* (**C**), and *Hey^UPcrIN2cr^* (**D**) mutants. In the double-deletion mutants, the Hey-positive cell population is greatly reduced compared to the single mutants. (**E**–**G’**): Sagittal views of stage 13 embryos. Anterior is up/left and ventral is left/down. Hey (green) is expressed in neurons of the developing CNS as well as in the deep internal area of developing midgut primordia in *Hey^UPcr^* (**E**) and *Hey^IN2cr^* (**F**). In the double-deletion mutant embryo *Hey^UPcrIN2cr^* (**G**), there is no Hey (green) in the developing midgut (arrowhead). The G inset displays the same image with Hey(green) and Pros (red) channels to show that the Pros positive EE population is present. (**H**–**J”**) Brain hemispheres, imaged from the dorsal (**H**,**I**,**J**) and ventral (**H’**,**I’**,**J’**) sides, and images of the VNC thoracic ganglia (**H”**,**I”**,**J”**) of Hey CRISPR mutants as designated. Hey (red) positive neurons are seen in every lineage marked by Ase-positive cells (NBs and GMCs) in both *Hey^UPcr^* (**H**–**H”**) and *Hey^IN2cr^* (**I**–**I”**) mutant lines. Intense Hey staining is evident in the optic ganglia as well. In the *Hey^UPcrIN2cr^* (**J**–**J”**) double mutant, in addition to the intensely stained MB Kenyon cells (yellow arrowhead in J), only a few other lineages in the central brain and in the VNC display Hey-positive neurons. Arrows in (**J**) indicate presumptive HeyIN2-negative lineages (based on their dorsal–posterior position, next to optic lobes). Scale bars: 48 μm (**B**–**D**), 90 μm (**E**,**G**), 80 μm (**F**), 70 μm (**H**,**I**), and 54 μm (**J**).

It was evident already during the course of the screening process that either one of the CRISPR deletion chromosomes can complement the *Df(2R)Hey^7552/6656^* mutant allele, as adults could be recovered over it. Thus, it was no surprise that the two different *Hey^IN2cr^* and the three different *Hey^UPcr^* mutant lines we established are all homozygous, viable, and fertile, suggesting that the overall Hey function is retained. Next, we examined the expression pattern of Hey to detect possible qualitative or quantitative differences to the wild-type one. In the embryonic CNS of both lines, Hey expression displays a wild-type pattern both in early (Supplementary Figure S9) and late stages (Figure 8B,C). Moreover, using the lineage specific markers Eve for aCC/pCC, RP2/RP2sib and AJ96-lacZ for dMP2/vMP2 sibling pairs of B/A-type neurons, we confirmed that Hey expression is in the “A”-type ones (pCC, RP2sib, and vMP2) indicating that in the CNS of either mutant line, Hey is normally activated in response to Notch (Supplementary Figure S9). Similarly, Hey seems to be normally expressed in the embryonic midgut primordia (Figure 8E,F) or in the third instar larva CNS (Figure 8H–I”) of both *Hey^UPcr^* and *Hey^IN2cr^* lines.

The above results support the idea of redundancy between the two Notch-responsive enhancer units, which we further investigated by generating a doubly mutant CRISPR line that combines both of the above deletions. This was achieved by inducing the HeyUP^cr^ deletion in the background of *HeyIN2^cr^* via CRISPR/Cas technology (see Methods) As all 6 Su(H) binding sites have now been removed, any residual Hey observed should be presumably expressed solely in the Notch-independent mode. The new *Hey^UPcrIN2cr^* line was homozygous lethal, exhibiting the characteristics of the *Df(2R)Hey^7552/6656^* and *Hey^f06656^* mutant phenotypes. Mutant embryos die at the end of embryogenesis, although a higher percentage (~40%) of hatching is observed compared to the null alleles (Figure 8A). The newly hatched larvae are sick and sluggish and die soon after, as first instars, but noticeably fewer of them succeed in going through the first instar molt and, in rare cases, they even reach the third instar. Looking at the expression of Hey in the CNS of these escapees, we realized that in addition to MB lineages (yellow arrowhead in Figure 8J), we also detect Hey-positive cells weakly expressing in several NB lineages. Actually, three of them seem to be the ones identified as HeyIN2-negative, based on their position (dorsal–posterior, next to the optic lobes) (arrows in Figure 8J). Almost no Hey-positive lineages were identified in the thoracic ganglia of VNC (Figure 8J”), while a reduced number of Hey-positive cells is observed in the optic lobes. In homozygous mutant embryos (Figure 8D), Hey expression in the developing CNS is now restricted in groups of cells along either side of the midline, a pattern which is reminiscent of the one observed in the *spdo^c55^* mutant [25]. This is expected, as this mutant displays only Notch-independent Hey expression, since Spdo is a Notch pathway component not needed in the earlier neuroblast lateral inhibition process; thus, the gross CNS anatomy is not affected by the mutation (in contrast to *Dl^−^ Ser^−^*). Finally, it is noteworthy that in the embryonic midgut primordia, we do not detect any Hey (Figure 8G), which is in agreement with our previous results that Hey expression in this tissue is exclusively Notch-dependent [27].

Collectively, all the above results strongly indicate that between the clusters of the Su(H) binding sites in the HeyUP and HeyIN2 regions, there is strong redundancy as regards the responsiveness of Hey expression to Notch signaling. Most importantly the double CRISPR mutant lethal phenotype clearly shows that the Notch-dependent mode of *Hey* gene expression is absolutely required for the viability of the organism.

## 4. Discussion

Hey is a Notch target in many asymmetric cell divisions, more specifically in the GMC to neurons division within the developing CNS [25] and the pre-EE to EE cells division within intestinal tissue [27]. On top of that, Hey possesses Notch-independent expression in the larva mushroom body lineages and in lateral embryonic VNC cell clusters [25]. ChIP data [37] and the identification of multiple high affinity Su(H) binding sites upstream of *Hey* transcription start and in the second intron of the gene provided a link between Notch activity and Hey expression and primed the experiments of this study. Using reporter transgenes, we validated both regions as enhancers associated with the *Hey* gene, that direct spatiotemporally overlapping expression patterns and that are partially redundant. They both support Hey expression in its Notch-dependent pattern, but only one, the HeyUP enhancer, drives expression in the Notch-independent subdomains. The fact that both enhancers drive expression in two apparently unrelated tissues is not so unexpected considering the evolutionary relationship between neurons and EE cells [46]. We also generated a *Hey* null allele and confirmed its recessive lethality at the embryo to L1 larva transition as previously observed for the *Hey^f06656^* mutant. Despite Hey expression in a large subset of embryonic neurons at the time of Notch-dependent fate acquisition, its deletion does not seem to affect asymmetric neuron fates, unlike a *Notch* mutation and similarly to the *Hey^f06656^* mutant [25]. If the lethality is not due to neuron mis-specification, it could be due to neuronal malfunction or even EE cell mis-specification, possibilities that have not yet been explored.

CRISPR deletion of the Su(H) binding site clusters in either enhancer has no effect on Hey expression or animal viability. However, a double-deletion eliminates all Notch-dependent expression and results in lethality only mildly improved compared to the null alleles—a few animals progress to the late larval stages before dying. We conclude that these two are true shadow enhancers that are sufficient to drive functional levels of Hey and encompass the entire Notch-dependent response, i.e., no other Notch-responsive shadow enhancer exists for *Hey*, which is also in agreement with ChIP data. Moreover, the lethality phenotype of the double CRISPR *Hey* mutant (*Hey^UPcrIN2cr^*) strongly suggests that expression of Hey in the Notch-dependent domains is the one strictly required for embryo viability as the *Hey^UPcrIN2cr^* mutants have intact expression of Hey in the Notch-independent domains. We mapped this expression to the HeyUP enhancer, and this may account for the double CRISPR allele’s mildly improved viability compared to the null. Alternatively, this mutant may retain a vestige of Notch-dependent Hey expression due to the undeleted single Su(H) site far upstream, in the 3′ UTR of the neighboring CG11191 gene or putative sites of more relaxed motifs (i.e KTGRRAA) found downstream of the HeyUP^cr^ deletion; this expression is not detectable by our antiserum in most experiments, although it is occasionally seen at very weak levels (e.g., Figure 8J larva). In any case, this is not sufficient to rescue the lethality of *Hey^UPcrIN2cr^* mutant.

As is the case for most shadow enhancers (i.e., [47]), the two Hey enhancers do not drive the exact same expression. In addition to the possession of the Notch-independent modules solely in the HeyUP enhancer, some additional differences were noted. HeyIN2 lacks expression in some L3 Hey-positive neuronal lineages, but it drives expression in “ectopic” cells, i.e., cells that do not express Hey protein. Most of these ectopic cells could not be trivially accounted for by the increased perdurance of GFP/β-gal relative to the Hey protein, as they belong to Hey-negative expression domains. Specifically, these are optic ganglia (presumptive lamina) cells, older Kenyon cells, sporadic mature larval neurons, embryo longitudinal glia, and ICP cells of embryonic midgut primordia. Most of these ectopic sites of expression (older Kenyon cells, mature larval neurons, embryonic glia) are retained upon point mutation of the four Su(H) binding sites of the Notch-unresponsive variant (*HeyIN2^mSALL^*), but the ectopic expression in the optic ganglia and the embryonic midgut ICPs disappeared, suggesting that they are regions that receive Notch input, but Hey ends up not accumulating. In support of this assumption, we have previously detected, reproducibly, very low Hey protein levels in 3–4 large nuclei of presumptive ICPs at the posterior midgut rudiment of stage 11 embryos [27]; thus, it is conceivable that activation of HeyIN2 in these cells might not be totally ectopic. In all the above cases though, suppression of HeyIN2 enhancer activation could be due to putative interactions with other enhancers (within the *Hey* locus or even more distant ones). An alternative scenario is that *Hey* is transcribed in some or all of these cells, but post-transcriptional processes (suppression of mRNA translation, mRNA/protein degradation, etc.) restrict Hey protein expression. In support to this notion, putative post-transcriptional regulation of Hey by TDP-43-like RNA binding protein [48] and putative post-translational regulation by the STUBL (SUMO-targeted UBiQUITIN LIGASE) protein degringolade [49] have been previously reported. 

Unlike HeyIN2, the HeyUP enhancer shows significant de-repression upon removal of its distal-most 400 bp, which contain all three Su(H) binding sites (HeyUP^NSH^ variant). In the embryo VNC, this is seen as doublets of neurons, only one of which is Hey-positive, probably due to expression in both siblings of an asymmetric division, e.g., the U/Usib neurons. In the larva, NSH de-repression is more widespread, evident in numerous secondary neurons that do not normally express Hey. This is not uncommon for Notch-responsive enhancers; other enhancers with strong activation input rely on their Su(H) sites to repress transcription in Notch-off cells, e.g., the *E(spl)m8* and *E(spl)mα* enhancers in pro-neural clusters [20] or the *vg*-boundary enhancer in wing pouch outside the DV boundary [50]. Su(H) has been documented to interact with numerous corepressors in the absence of N^icd^, which displaces the corepressors and acts as a co-activator [18]. Whereas deletion of the Su(H) sites resulted in de-repression in the HeyUP^NSH^ reporter constructs, the same effect was not seen at the protein level in the endogenous locus in the *Hey^UPcrIN2cr^* allele, which also lacks Su(H) binding sites. One explanation may be the fact that a single HeyUP Su(H) site has been left intact in the CRISPR allele, and it may suffice to repress all this ectopic expression. Retention of the distal part of the HeyUP enhancer (311 bp) may also explain the occurrence of the Notch-independent embryonic lateral VNC pattern in the *Hey^UPcrIN2cr^* allele, which is absent in the HeyUP^NSH^ reporters. 

In summary, Notch-dependent expression of Hey is supported by two CRMs which share several features of shadow enhancers [51,52,53]. One is located within *Hey’s* second intron, and the other is far upstream in the 3′UTR of the neighboring gene; both drive transcription in response to Notch and in patterns similar to each other and to endogenous *Hey*. Shadow enhancers can be redundant at one developmental stage while having non-redundant roles in other stages/tissues [54]. They may also share slightly different roles in regulating gene expression, as evidenced when deleting one or the other [55]. Contrary to the above, the *Hey* enhancers are redundant in regard to their Notch dependence in the majority of Hey-positive cells in the same tissues and at two different developmental stages (embryo, larva). Moreover, CRISPR deletion of either one does not affect the viability or the expression of Hey in specific CNS lineages and the midgut EE cell subpopulation of the embryo. However, whereas the HeyUP and HeyIN2 enhancers act as shadows of one another, detailed analysis of the isolated enhancers has revealed important differences, both in their response to Notch and in their ability to express in Notch-off regions, issues that require further investigation. The new genetic lines analyzed in this study provide important tools in the study of Hey expression as well as in the characterization of the functional role of Hey in the developing neural and intestinal tissues.

## Figures and Tables

**Table 1:** List of fly lines generated in this study.

Study of the Hey Upstream Region
attp40:* HeyUP^FL^-nbgal*
attp40:* HeyUP^FL^-n GFP*
attp40: *HeyUP^2+3SH^-nGFP*
attp40: *HeyUP^NSH^-nGFP*
**Study of the Hey intron 2**
attp40:* HeyIN2-nGFP*
attp2:* HeyIN2-n bgal*
attp40:* HeyIN2-n bgal*
attp40:* Heyin2^mSALL^-nGFP*
attp40:* Heyin2^mSALL^-nlacZ*
**Hey mutants**
Df(2R)*Hey^7552/6656^/CyO twistG > UASGFP*
*Hey^UPcr^*
*Hey^IN2cr^*
*Hey^UPcrIN2cr^/CyO,wglacZ*
*Hey^UPcrIN2cr^/CyO,* *twistG > UASGFP*

**Table 2:** List of primers.

Name	Sequence (5′ to 3′)	
	I. **Cloning and PCR**	
**FOR Heyup**	CAGAATTCCGGCGGCTTTGTAACCTCCACTTTGCG	[−2289 to −2264]
**REV Heyup**	CTGGTACCGAGGCAGATGCAGTTGGCGGCTAG	[+356 to +380]
**heyPf-E1**	GGAATTCGTGTGTATGTGTGTGCGGC’	[−2119 to −2101]
**UPFip**	GCGGAATTCCCTCCACTTTGCGTCCAGCGATA	[−2275 to −2252]
**UPRip**	GCGGGTACCTTGACTATAATGGAGCCCCGTATCAA	[−1656 to −1631]
**UPMB-F**	GCGGAATTCCCTCCACTTTGCGTCCAGCGATA	[−1895 to −1871]
**CDS-F**	CTGAGCTCATGGATCACAACATGCACGTCAATG	[+481 to +496]
**heyIr-K1**	GCGGTACCCTGCAACAAGATACGAGGAGG	[+2089 to +2109]
**heyIf-X1**	CGCTCTAGAATTACCAAGCCCACTTGAGC	[+1376 to +1395]
	II. **Site-directed mutagenesis**	
**1st Su(H)-NruI**	CTAAACGCAAGCCGCTGTCGCGAACAATTGAGTAACG	
**2nd Su(H)-StuI**	GTTGTCCCGAACAGGGTCGAATTGAGGCCTAATTATGATTGC	
**3rd Su(H)-SmaI**	CTCGAATCTCGGGGCTCCCGGGGCTCAGTAGC	
**4th Su(H)-ApaLI**	GGGTTGTTCTTTATGCTCGTCGCGTGCACCCAGCTATTTTAATTG	
	III. **FLP/FRT deletion mutant**	
**f07552GP fwd**	GGTGCCACCATGAACCAAACT	upstream of *WH f07552* (+)
**f06656GP REV**	TCGCGCACACGATAAGTCAC	Hey exon1(−) [+308 to +328]
**Tn1 REV**	TCCAAGCGGCGACTGAGATG	WH5′ plus-left primer [31]
**Tn2 FWD**	CCTCGATATACAGACCGATAAAAC	WH3′ plus-right primer [31]
	IV. **CRISPR deletion mutants**	
**INT2 5** **′** **gRNA**	CTTC GAGCAAATAGAGGGTTAACT AAAC AGTTAACCCTCTATTTGCTC	
**INT2 3** **′** **gRNA**	AAAC GAACTGGGATTAGACAGCTC CTTC GAGCTGTCTAATCCCAGTTC	
**UP 5** **′** **gRNA**	CTTC GTGCGGGTCGTGCGCCGGAA AAAC TTCCGGCGCACGACCCGCAC	
**UP 3′gRNA**	CTTC GCTTGATTTGGGGTGGGTTC AAAC GAACCCACCCCAAATCAAGC	

## Data Availability

The raw data supporting the conclusions of this article will be made available by the authors on request.

## Supplementary Materials

The following supporting information can be downloaded at: https://www.mdpi.com/article/10.3390/genes15081071/s1, Figure S1. EVOPRINTER analysis of Hey locus. Figure S2. Hey enhancers are active in larval CNS. Figure S3. Ectopic activation of HeyIN2 enhancer in REPO positive embryonic glia. Figure S4. Hey enhancers’ expression in the larva CNS. Figure S5. MARCM analysis in larva CNS. Figure S6. FLP/FRT induced Df(2R)Hey7552/6656 mutant. 7552/6656 Superscript. Figure S7. Su(H) binding clusters of Hey locus. Figure S8. PCR screening for CRISPR/Cas9 induced Hey deletion mutants. Figure S9. Hey expression in the CNS of Hey CRISPR mutant embryos.
