# Supplementary material for: Notch-Dependent Expression of the Drosophila Hey Gene Is Supported by a Pair of Enhancers with Overlapping Activities
Source: Genes (Basel). 2024 Aug 14;15(8):1071. doi: 10.3390/genes15081071 (PMC11353301; doi:10.3390/genes15081071)

Supplementary Figures

Figure S1. EVOPRINTER analysis of *Hey* locus

The *EvoPrinter* output represents the *Drosophila melanogaster* sequence from the *Hey* locus used for *in silico* evolutionary analysis. Lower case base pairs are not conserved in all eight species (*Drosophila melanogaster, D.simulans, D.sechellia, D.erecta, D.yakuba, D.ananassae, D.pseudoobscura and D.persimilis*) whereas upper case base pairs are evolutionary conserved. *Hey* gene is transcribed from the complementary sequence of the one presented, in the orientation shown by the arrow on the right side of the sequence. Exons are in red letters and UTRs in black. The 3'UTR of the neighboring *CG11191* gene which is transcribed in the same orientation as *Hey,* is shown with pink letters and its termination codon in turquoise highlight. The high affinity motifs for Su(H) binding present in *Hey* intron 2 and in the 3'UTR of CG11191 are highlighted yellow and putative less affinity motifs are highlighted in grey.

Figure S2. Hey enhancers are active in larval CNS.

Images presenting X-gal activity staining in 3rd instar larval CNS of HeyUP^{FL}-bgal and HeyIN2-bgal transgenic lines monitored in two different time points as designated.

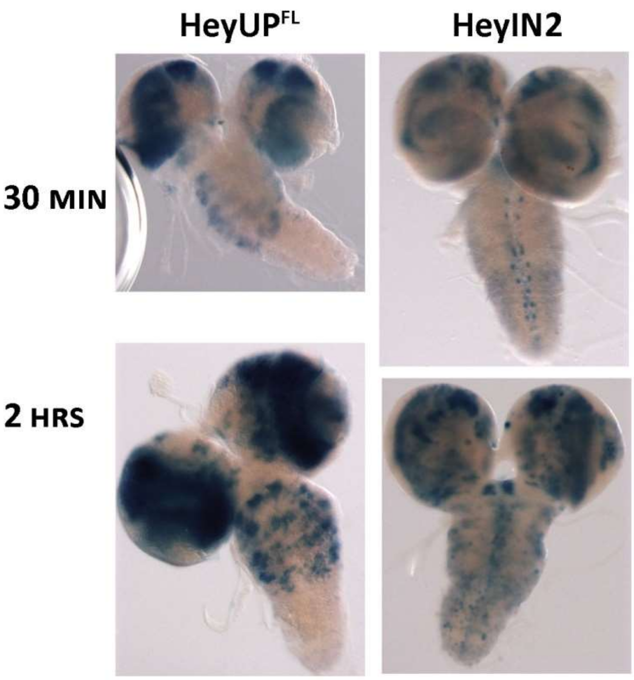

Figure S3. Ectopic activation of HeyIN2 enhancer in REPO positive embryonic glia

A-A'": Stage 13 embryo CNS stained for HeyIN2-bgal (green), Hey (blue) and Repo (red). Arrowheads (A'-A'") point to Repo positive glia nuclei that are also positive for Hey and HeyIN2-bgal while arrows (A, A") indicate Repo positive, HeyIN2-bgal positive glia that are Hey negative. Scale bar: 30µm. **B-B'":** Sagittal view of a stage 16 embryo and projection of confocal sections from deep layers close to midline showing ectopic expression of HeyIN2-bgal reporter in Repo positive longitudinal glia (large arrows) that are Hey negative. Scale bar: 50µm

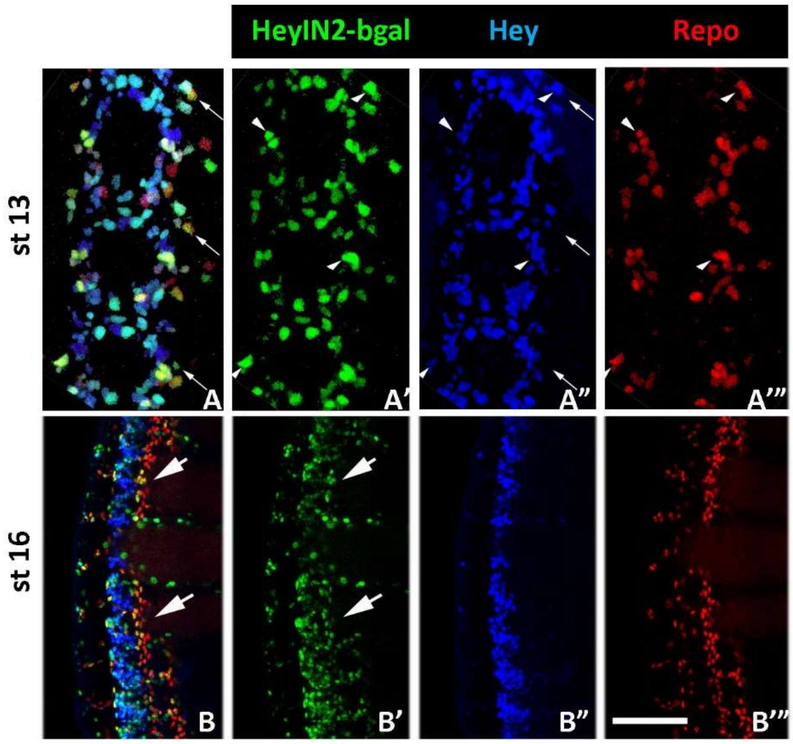

Figure S4. Hey enhancers' expression in the larva CNS

Projections of confocal optical sections of brain hemispheres from larvae bearing HeyUP^{FL}-GFP and HeyIN2- βgal transgenes (double transgene genotype) stained for Hey (blue), GFP (green, for HeyUP^{FL}-GFP) and bgal (red, for HeyIN2- bgal). **(A-A')** Dorsal view of 3rd instar larva brain showing MB lineages (4 small yellow arrows) area. HeyIN2-bgal positive cells (A'- βgal channel) are around HeyUP^{FL}-GFP positive ones. White arrows point to two lineages that are HeyUP^{FL}-GFP positive and HeyIN2-bgal negative. **(B-B')** Higher magnification image showing MB lineages of 3rd instar larva brain where the two discrete transgene expression domains corresponding to the differential activation of HeyUP^{FL} and HeyIN2 enhancers are nicely monitored simultaneously. Hey (blue) colocalizes with HeyUP^{FL}-GFP (green) in young Kenyon cells (B) that are surrounded by HeyIN2-bgal positive older ones (B', Hey and b-gal channels only). **(C-C')** Notably, in double reporter 2nd instar CNSs only Hey and HeyUP^{FL}-GFP expression is evident in the

newborn Kenyon cells and no expression of HeyIN2-bgal is observed. Asterisks in C' (bgal channel) point to the MB area that is devoid of bgal staining. **(D-D'")** Ventral view of 3rd instar larva brain. Note the HeyIN2-bgal ectopic expression in the OL, in a ring of cells (yellow arrowhead in D, D'") that are Hey negative (D'). Small white arrows (D', D'") point to more b-gal positive / Hey negative cells of HeyIN2 ectopic activation.
Scale bar: 70μm (A, D), 30μm (B), 40μm (C).

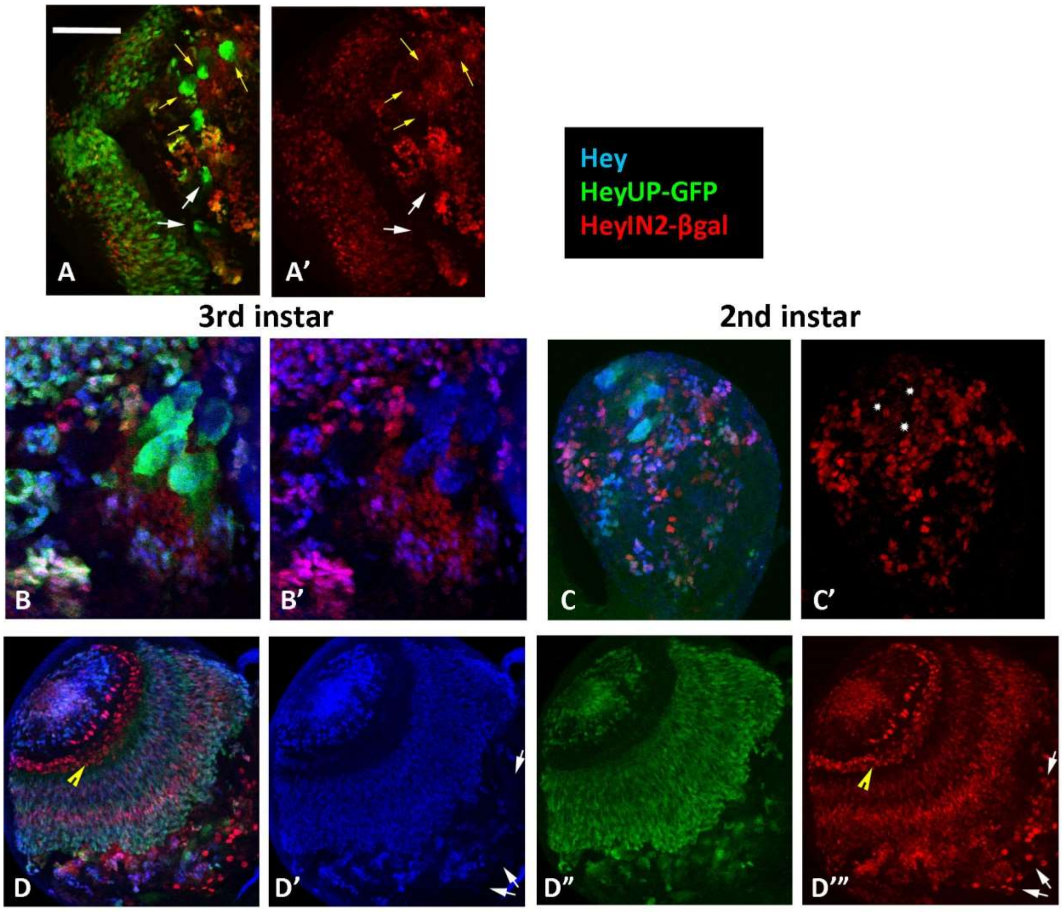

Figure S5. MARCM analysis in larva CNS

Examples of GFP-marked (green) mutant *Dl⁻ Ser⁻* clones stained for Hey (blue) and bgal (red) that presents HeyUP^{FL}-bgal or HeyIN2-bgal expression as designated. All mutant clones that are devoid of Hey positive cells they are also bgal negative.

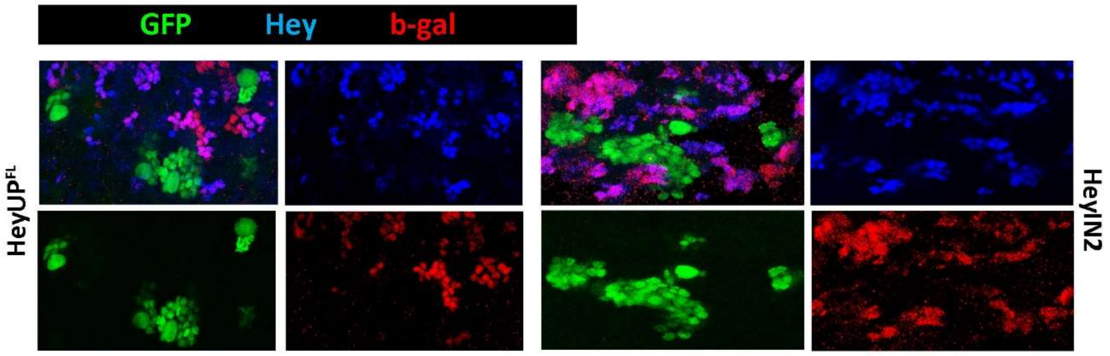

Figure S6. FLP/FRT induced *Df(2R)Hey$^{7552/6656}$* mutant.

A: Diagram depicting *Hey* genomic locus and the neighboring *Dic3* gene as well as the positions of the two *piggyBac* insertions WH(+)f07552 and WH(+)f06656 bearing an FRT site. Arrowhead designates the FRT same orientation in both elements. Primers Tn1 and Tn2 for WH(+)f07552 and WH(+)f06656 respectively as well as the corresponding genomic primers GP7552 and GP6656 designed for the (A) and (B) PCR reactions are designated as arrows of opposite orientation. **B:** Image of an agarose gel, example of the genomic PCR screening for recovering a Hey FRT-based deletion mutant. Seven different lines are analyzed for products 866bp and 720 bp from both (A) and (B) PCR reactions respectively. The deletion has been recovered in all lines except N5.

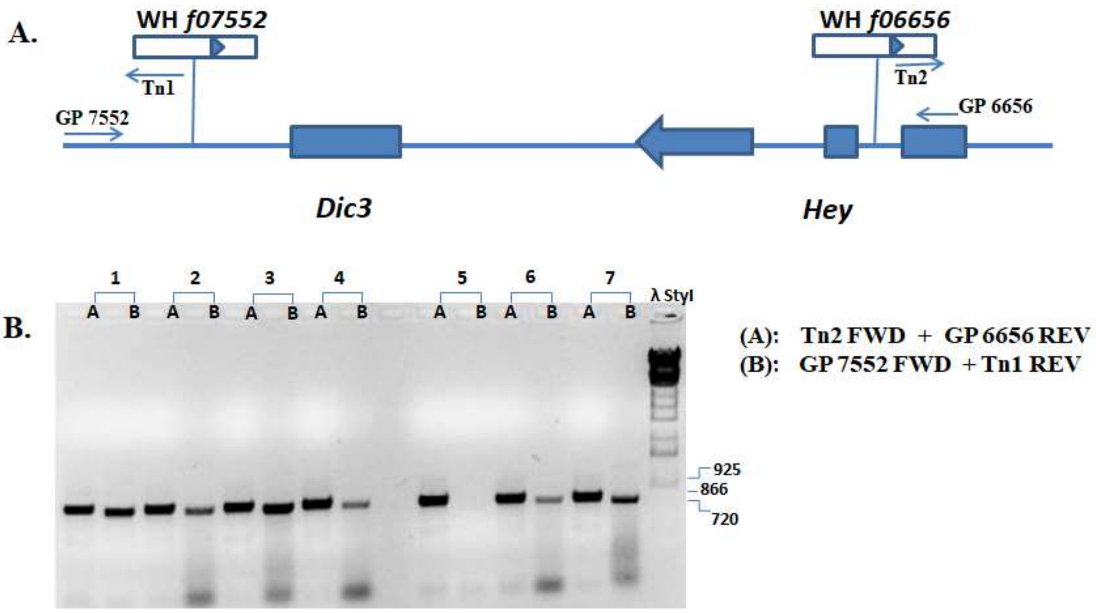

Figure S7. Su(H) binding clusters of *Hey* locus

Sequence from two genomic regions displaying the Su(H) binding sites in the vicinity of *Hey*. The cluster of the upstream region includes 3 motifs (red capital letters) within the 3'UTR (upper case letters) of the *CG11191* locus downstream of the stop codon (green underlined letters). Lower case letters are intergenic region between *Hey* and *CG11191*. The *Hey* intron 2 cluster includes 4 motifs (red letters) within the second intron of *Hey* presented with lower case while exon2 and exon3 sequences are shown with upper case letters. Boxed sequences, highlighted yellow, represent the gRNAs (with underlined PAM sequence) used for CRISPR/Cas induced *Hey* deletion mutants. The primers used in the genomic PCR screening for the recovery of either deletion are designated with blue arrows

Hey upstream Su(H) cluster

TGAGACCGGAAACGGACACTCGGCGGCTTTGTAACCTCCACTTTGCGTCCAGCGATAGAGCACGAAAACCAGTCT**TGTGAGAA**T
TACCTACATACGAGTATATTATACATATATGTAATCGAGTATTTGAGTCGGTAGACCGCAATTTTCATTATAGAAAGTTGTTTTCGAGA
ATATCCATTAACAATAAAGTGTGTATGTGTGTGCGGCGCCATACCTTTGGCTCCATACCTTGAGTAATGCACTTCAGAATGCGAATAC
GAATGCGAATGCCCTGTGCCCCGTGGGTGGGAGTAAGTTCATCGCCCACCCATCCATCCATCGAT**CCATTCCGGCGCACGACCCGC**
ACGTGTGCTGGACGCT**CGTGAGAA**TCCGTAGACCTTTGGCT**TTCCCACg**agccacggctgcgatttcgtgggttcgtcccgttgtcggtggtgcaac
ttgcccgccactattcatc**acttgatttggggtgggttccgg**cgattcggagcgagtttgtcccacgctggcactgccactggcattggccattagacattaggcattttca
ctttgctgcttgccgcactcgaaacctgagcgacagcatgccgaatcaaatgaaaagcactttttataattttcgattttgatgtacgtttttgaaattgatacggggctcca
ttatagtcaagcttacgcctcgaccatcgaaattaatcgag [+1599nt to Hey Exon1(+1)]

Hey Intron2 Su(H) cluster

CAGTCTAAAAgtaagtttaccaagcccacttgagctagcttaaagaggttttaaaaagcaaataggctagctgcaccacagacctcctcactgaagttaaacgct
tctatgggtcattaatcacaatag**gagcaaatagagggttaacttggg**cagccaactaagttggttaactaaaagaagtaaagtgtaaagaaagtaatttcttatttc
gacagttaatattacattagggcccatttagcgcactaattaatcaaggcatatctgacatacatatgaacttcaatacattataaaaaattggaatgggaaaaactgc
aagcagaatccctaaacgcaagccgctgtgggaaacaattgagtaacgatattaaatacaatatgcggcaaattatcacaaatctgggtaaacaatcctgacctgtt
gtcccgaacagggtcgaattgtgggaaaattatgattgccgtaggcaaagcaggaccaccaccaccagcgccagcctagcgctcaagtgaatctcgaatctcgggg
cttcccacgctcagtagcggcaccaccacgatggcaaccttgtagccaggcgcgggctttcacccccgatagccacctcacaacaagccatcccgccgaaggacat
gggggttgttctttatgctcgtcgcgtgggaacagctattttaattgtttg**cctgaactgggattagacagctt**attgactttctccacctcctcgtatcttgttgcagCTC
TCGACTCGCTTAGCTATGATCCGCAGCGAGTGGCCATGG

Figure S8. PCR screening for CRISPR/Cas9 induced *Hey* deletion mutants.

Representative gel images of genomic PCR (parental crosses). **A. HeyUPcr:** primers UPFip and UPRip, amplify a 646bp product unless a CRISPR deletion event is induced producing a 497bp fragment; **B. HeyIN2cr:** heyIf-X1 and heyIr-K1 primers at the 5'end and 3' end of intron2 amplify either a 734 bp wild type fragment or a 181 bp product representing a CRISPR event; **C.** *y M{nos-Cas9.P}ZH-2A w** strain bearing homozygous HeyIN2cr deletion identified by a 1075bp fragment instead of the wild type 1628bp, product of CDSF (located at the beginning of *Hey* coding region) and heyIr-K1 pair of primers; **D.** Induction of the HeyUPcr deletion in the background of the *y M{nos-Cas9.P}ZH-2A w* ; Hey*^{In2cr} strain

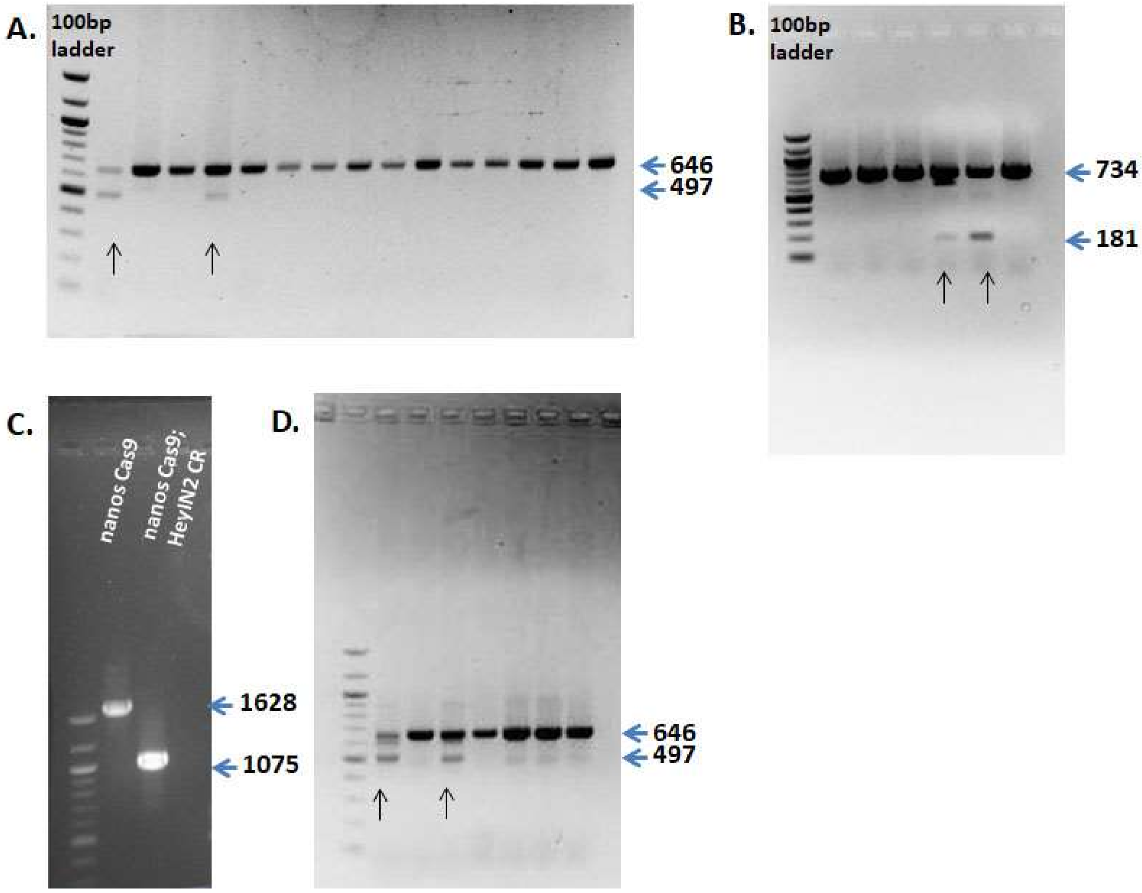

Figure S9. Hey expression in the CNS of Hey CRISPR mutant embryos.

A: Stage 11/12 embryos of Hey^{UPcr}, Hey^{IN2cr} and $Hey^{UPcrIN2cr}$, mutants as designated. In deep focal planes (montage in Hey^{UPcr} image), Eve (red) marks aCC/pCC pairs together with some U neurons (arrowheads) and RP2/RP2sib pairs (arrows). Hey expression (blue) in Hey^{UPcr}, and Hey^{IN2cr} is retained in the pCC, Us and RP2sib as in the wild type. In the $Hey^{UPcrIN2cr}$ double mutants, the Hey positive cell population is a lot reduced compared to the single mutants and the Eve lineages are devoid of Hey expression (yellow arrowheads). Anterior is up. Scale bar: 40μm. **B:** Stage 11/12 embryos of Hey^{UPcr}, Hey^{IN2cr} and $Hey^{UPcrIN2cr}$, mutants as designated bearing the AJ96-bgal transgene in the 3rd chromosome. Anterior is up. b-galactosidase (green) marks vMP2/dMP2 neurons and vMP2 (anterior cell) is also marked by Hey expression (red) in Hey^{UPcr}, Hey^{IN2cr} mutants as in wild type.

In the $Hey^{UPcrIN2cr}$ double mutants Hey is not expressed in this lineage at all. Scale bar: 50μm

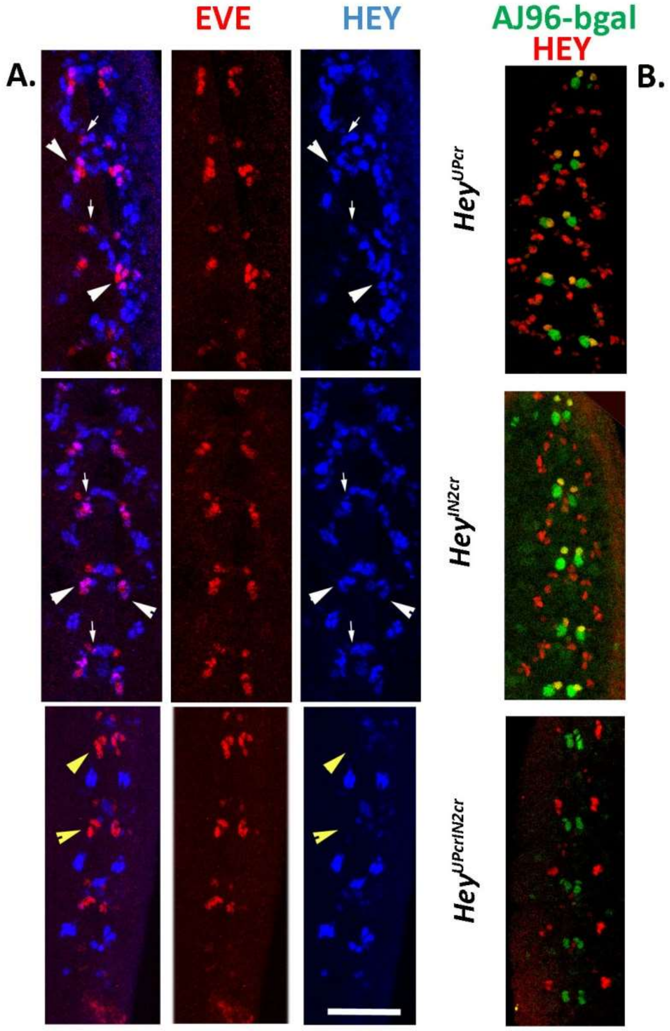

Supplement: Supplementary file 1 [file genes-15-01071-s001.zip › genes-3113946-supplementary.pdf]
