# Supplementary material for: Notch-Dependent Expression of the Drosophila Hey Gene Is Supported by a Pair of Enhancers with Overlapping Activities
Source: Genes (Basel). 2024 Aug 14;15(8):1071. doi: 10.3390/genes15081071 (PMC11353301; doi:10.3390/genes15081071)
Supplement: Supplementary file 1 [file Suppl FIGURE S1.pdf › Suppl FIGURE S1.pdf]

Capital letters represent nucleotides in the *D.melanogaster Hey* reference sequence that are conserved in the *D.simulans, D.sechellia, D.erecta, D.yakuba, D.ananassae, D.pseudoobscura* and *D.persimilis* orthologous DNAs.

cgtctgtatgaacgtcgagatttcacgaaatataacaactagaaaggctttcctgatccgaatatcaattatcaagcgagtttcaacatat
tcacttatacactcatgcgTTTATTTACAATAGatTCACAAAgtgTTCaaagttatagttatcatagggtataacttaagcttaaaggctt
acgggactagctAATCTCActgcgttgtttctccatttttttctctctgtgtaggatgcgaaACTTAagACTATCACACTtGGtctgctct
GACTagaggttgaaatatcccttctgaggcttatgcccacaatcaaTAGGCCATcTCgGCgCCCCAaggacgaTAGGGcTTtGCtCCGTTG
GGaGTGGCgTAcTGcAGcACaCTgGaGTTGTTGTAgTCCTGgCCtggcagtccgctgaccGGaTAcTGaGGaGCaGcaCTaTAgCTaAGGc
tatcggaacctatgGCcGCtGGacgattGCTGTTGGTcAGcTCtATGTAGGTGGGaacCTGcTGCTcGGaaTGcactgcgctgtggtcgtg
ggtatagTGtTGCTGCTGcgtcggctgaggtgttgtctgcgtccgtTGCTGTTGCTGCTGaTGcTGCTGCTGttggtgttgctgctgctgc
tgctgttgctgctgctgtTGcTGCTGCTGtTGgCTGCtgctgtcagagtggagatcatggctgggcagcgattcagttacggcacttccac
ttgtccgcgagacactggtgcgtcctcccagttgctgagcctgctgaGAgGGCGAgGCaCTtagtgtggGGTAGCTGGAcACATAGGcacC
CGGGTTggcTGgcgccgcaTAGcTCTGGTAgGGAGcaGCcGCACAgtTtGGctgatagccggaggagCtgGGagcaGcCggaGaCCAGCCa
cccGGACTGgCgCAgcTCTtgGCcGAcAGtTCgCGCTGCTGcACaAAGTACTGgAGGTGgGACATaAGGCGCAgGCGCAGaGGATCCTGaA
TgTCCATGCCCTCGATGGTgACcAaaTAgCGGGCCACcTCtGCGGCGCACTCgCGGAAcCCaATgaTaTGGTAGTCCATGGCCACtCGCTG
cGGaTCaTAGCTaAGCGAgTCgAgagCTGCAACaAgatacgaggAGGtGGAGAAAGTCAATAAgCTGTCTAATCCcagttCaGGcAAACAA
TTAaAATAGcTgTTCCCACGCGACgagcataaagaacaacccccatgtccttcggcgggatggcttgttgtgaggtggctatcggggggtg inS4
aaagcccgcgcctggctacaaggttgccatcgtggtggtgccgctactgAGCGTGGGAAGCCCCGAGATTCgAGaTTCACTtgagcgctag inS3
gctggcgctggtggtggtggtcctgctttgcctacGgcaAtCATAaTTTTCCCAcaAttcgacCCTGttcGGgACAACAGGTcAGGAtTGT inS2
TTACcCaGATTTgtgATAATttgccgcatattgtatttaatatcgttactcaattgtttcccacagcggcttgcgtttagggattctgctt inS1
gcagtttttcccattccaatttttataatgtattgaagttcatatgtatgtcagatatgccttgattaattagtgcgctaaatgggccct
aatgtaatattaactgtcgaaataagaaattactttctttacactttacttcttttagttaaccaacttagttggctgcccaagttaaccc
tctatttgctcctattgtgattaatgacccatagaagcgtttaacttcagtgaggaggtctgtggtgcagctagcctatttgctttttaaa
acctctttaagctagctcaagtgggcttggtaaACTTACTTTTaGAcTGcAgacTCTTgAGaTGCTCCACgGTcAGCTGaAGgATtTCgGC
cTTcTCcAgCTTGGCgGAtCCcTGcTTCTCgTAgGCgGAgGGCACCAGgCgCTTgAGTTCGGTgAGcGAgGAGTTGATcCGATCcCGcCGC
TTCTTCTCgATcACtCCgCGtCgCTTcTTGCGGCTCATcAGtTGGCAGCTgcCcGgcTcACTGGGcGAGATTCTaatggaacagaggaaag
ggtttctattagctatggattgaagttaatgatagattcttaatcttaatatcttaatctttcatagactgaaaaacactctatggtgtta
ctaaaaaaacctctaagttacagaacattttaaaatctatcttttcttaaaaccctatagtttagtgatttatattttgatggtttatcag
caactttgcgttaagtggagttatccatgacttcgattcactaagtaaatatcatgacgcactatttttttagcaacataaaacgtttgc
acatcgcatggtattttcaaaggtttcattttacaagacaactcctaacttgggaactgcgaatagctttttgtgcactaaattgaaaaac
aaattttgataagaagtcacgagtacgcactgttccttggaggactcctcggagtagagatcgtcgcagtcgctctcggaaagcgtccgct
tcaggctgccgatgccgtggctatggccctggctatgcccgtggctgtggttgctgtgggcgctgtggtggctctgcggcggcggcaccca
gtggctctggggtgtggtggcggtggcgccgggcatgaccacgccgggtccggcggcgtagccccagtgatgcagcgatggcgcattgacg
tgcatgttgtgatccattgtgtgtggtgaccagtgtttactcgcTGcTcCTGCGCACTTGaCactactgtgttgatgttgctgttgctgag
ctgtagctttgcgtgctgctagttgcagaggcagatgcagttggcggctagaccgggctgGCGGAGCTaGtTTGAgCACGTGACTTATCGT
GTGCGCGAACGGCGTTCGACTGATTcTGtAGCACACGtCgAGCGGACGACCTGCCctcCgCTGCCGCCGCTCAACCCCTTTtTACCGCCtg
cGGCAgcCGtCCAATAAGATGCAGCAGCAACCACCCCcTGGCaACGcGcagcgcgtgcttgccgagattggggtaggaaacgggaagcaaa Hey
catattcgccgcgcggcgatcgccgccaagtgagcaagtgagagtgtgcgacaaggagcgaccagcgccggcaaatgagcaagcctctgtc
atcgattcatccgattgtatcgcccgaccatctgatatagcctttcagcagcctctaaagcatctaaagcatctaaagcatctgcagcacg
gcctttAATCggCGCCGTGGGCGacaaactggcggcGTCCGCCGtCCACTTGtTtGCcGCtggagcggCTGcaCCCGtccatggAActcaA
ACTGGAGCACGCGCCGGTCGTGTGGCatcgcgtggcgccgggtaaaGCCCCAACAAGTGCAACaGCCGcCGtTGACCTcTATCTgtgcagt
gggacgcgtgctggtgggtggcccacacAgTTCtAGGTCAGTGccGgCTCAAGGttgcggcatcccgggtccggaatgggaacgggaacat
ccacaggtgcagaagcaggagcaggagacgagataacaacgactgcggcagcaggtgaccgaagttgacctagaaattgcccgaggtaaaa
tcgacttgtttacctagaaatatgctaatgcggaaataattcactttaatgttatcgaaagcagcgtttcatcacaaagggatataaggaa
cttggatggcgacggtaaaacgtaaatatgttgtacaattttacgaaaaagcagacgtgagctttatctttttctttatctttttgatttg
ccattataaactcttagatgcgctttgtcactgcatttcgaatattcacatgatttaatccattctaaatttttttgctgaattggctaatc
ttataacttccttaattgttccttgtaattgtatgaacgctgagctctcgggaactataaaagcatgaatgtaaaactagttactaggatt
aagagctagattgttacagaagattgcaacgcctactcttaaggacacaaaccggctaaaactgccacgtccatacttttgcaaaattgtc
atatttttaaaaatattttggccactcccagtctaatggccacaagctgtcaatctaaaacttttgaaaaatgttttgatatttttgatt
tcactattttttttgacagctttgatcgctattccaccaaaatttgtcagtttttctaatgattttacaagctgagatagttaacatcgaa
attaacgttctgtctgttttggttaatgtttccccattaactttaatgtaatatattttgacagtattgtgttcataaattcaatataaca
agctgatcaatgtcaatattacctataaattttattatgataaactatttcatccaaaatttcccattatttcttatatcagccgcacgga
atgtgtgtttaaagctagcagtagattaaaaacaattaaaatgcgaatgaaaaacagtatacggtggagctgatatatttacatatatatg
tacgttgtggtttgacataactttccctaataaaaactatacttgaattatcgttatgcaaaaataattcttctgtagatatctatgttaa
aatgttcatttcaactcctttgtcctgtcaccctaaagaatcccaatattgctatatgaatccctatactattgtatatacttttatatcc
ctttactattatatgttgcaatttcgcatataactggaaatatcattagttattctcccccacaaaatgatttagtgtagcatttctccca
ttttctcgattaatttcgatggtcgaggcgtaagcttgactataatggagccccgtatcaatttcaaaaacgtacatcaaaatcgaaaatt
aTAAAAaGTGCTTTTcATTTGATTCGGCATGCTGTCGCTCAGGTTtcgagtgcggcaagcagcAAAGTGaAAATGcctaatgtcTAATGGC
CAATGCCAGtggcagtgCCAGCGTGGGACAAaCtcgctccgaatcgccGGAACCCACCCCaAATCAAGTGgATgaATAGTGgcgggcaagT
TGCACCACCGacaacgggacgaacCCacgAAATCGCAGcCGTGGCTCGTGGGAAAGCCAAAGGTctacGGaTTCTCACGagcGTcCAGcaC upS3, upS2
acGTGCgGGTCGTGCGCCGgaatggatcgatggatggatgggtgggcgatgaacttactcccacccacggggcacagggcattcgcattcg
tattcgcattcTGAAGTGCATtacTCAAGGTATGGAGCCAAAGGtatggcgccgcacacacatacacaCTTTATTgTTAATGGAtaTTCTc
gaaAACAACTTTCTATAATGAAAATTGCGGTCTaCCgactcaaatactcgattacatatatgtataatatactcgtatgtagGTAaTTCTC upS1
ACaagactggttttcgtgctctatcgctggacgCAAAGTggaggttacaaagccgccgagtgtccgtttccggtcTcAGAAGCTCcTAAGG
CAAAgATTgcagttgtttccgcctactgcattgtcgtgt
